# Plastid translation is essential for lateral root stem cell patterning in *Arabidopsis thaliana*

**DOI:** 10.1242/bio.028175

**Published:** 2018-01-24

**Authors:** Miyuki T. Nakata, Mayuko Sato, Mayumi Wakazaki, Nozomi Sato, Koji Kojima, Akihiko Sekine, Shiori Nakamura, Toshiharu Shikanai, Kiminori Toyooka, Hirokazu Tsukaya, Gorou Horiguchi

**Affiliations:** 1 Research Center for Life Science, College of Science, Rikkyo University, Toshima, Tokyo 171-8501, Japan; 2 Center for Sustainable Resource Science, RIKEN, Tsurumi, Yokohama, Kanagawa 230-0045, Japan; 3 Graduate School of Science, Kyoto University, Sakyo, Kyoto 606-8502, Japan; 4 Department of Life Science, College of Science, Rikkyo University, Toshima, Tokyo 171-8501, Japan; 5 Graduate school of Science, The University of Tokyo, Bunkyo, Tokyo 113-0033, Japan; 6 Okazaki Institute for Integrative Bioscience, National Institutes of Natural Sciences, Okazaki, Aichi 444-8787, Japan

**Keywords:** Lateral root, Plastid, RFC3, Ribosome, Spectinomycin, Stem cell

## Abstract

The plastid evolved from a symbiotic cyanobacterial ancestor and is an essential organelle for plant life, but its developmental roles in roots have been largely overlooked. Here, we show that plastid translation is connected to the stem cell patterning in lateral root primordia. The *RFC3* gene encodes a plastid-localized protein that is a conserved bacterial ribosomal protein S6 of β/γ proteobacterial origin. The *rfc3* mutant developed lateral roots with disrupted stem cell patterning and associated with decreased leaf photosynthetic activity, reduced accumulation of plastid rRNAs in roots, altered root plastid gene expression, and changes in expression of several root stem cell regulators. These results suggest that deficiencies in plastid function affect lateral root stem cells. Treatment with the plastid translation inhibitor spectinomycin phenocopied the defective stem cell patterning in lateral roots and altered plastid gene expression observed in the *rfc3* mutant. Additionally, when *prps17* defective in a plastid ribosomal protein was treated with low concentrations of spectinomycin, it also phenocopied the lateral root phenotypes of *rfc3*. The spectinomycin treatment and *rfc3* mutation also negatively affected symplasmic connectivity between primary root and lateral root primordia. This study highlights previously unrecognized functions of plastid translation in the stem cell patterning in lateral roots.

## Abstract (precis)

**Summary:** Successful plastid gene expression is required for stem cell patterning in lateral root primordia as revealed by genetic and pharmacological impairment of plastid translation.

## INTRODUCTION

The plastid evolved from a symbiotic cyanobacterial ancestor and is an essential organelle of plants as the site of a number of metabolic reactions represented by photosynthesis in chloroplasts. The plastid has its own genome and gene expression machinery. Plastids and the nucleus communicate with each other to regulate cellular physiology and plastid functions, especially those related to photosynthesis. Mutations or treatment with drugs that interfere with metabolic pathways and gene expression machinery of chloroplasts evoke retrograde signaling and suppress the expression of photosynthesis-associated nuclear genes (Bobik and Burch-Smith, 2015; Kleine and Leister, 2016). Impaired translation in chloroplasts is a trigger for retrograde signaling (Tiller and Bock, 2014). Recent studies in *Arabidopsis thaliana* (hereafter, Arabidopsis) have suggested that plastids also have a role in particular developmental events, e.g. flowering time, leaf adaxial-abaxial patterning and callus formation, in shoots of higher plants (Moschopoulos et al., 2012; Tameshige et al., 2013; Mateo-Bonmatí et al., 2015; Wang and Dehesh, 2015; Wilson et al., 2016). In addition to the green chloroplasts found in photosynthetic tissues, non-green plastids are found in cells of heterotrophic organs, including roots (Robertson and Laetsch, 1974; Kobayashi et al., 2012); however, the role of translation in non-green plastids in root development is largely unknown.

We previously reported that a *regulator of fatty acid composition3* (*rfc3*) mutant of Arabidopsis strikingly forms abnormal lateral roots (LRs), in which function and stem cell patterning of the root apical meristem are completely disrupted or severely compromised (Horiguchi et al., 2003). The *RFC3* gene (At3g17170) encodes a protein harboring a plastid-localization signal and a bacterial ribosomal protein S6 (bRPS6)-like sequence (Horiguchi et al., 2003). There is an authentic *PLASTID RIBOSOMAL PROTEIN S6* (*PRPS6*) gene (At1g64510), of which an ortholog in spinach was identified by a proteome analysis of the chloroplast 30S ribosomal subunit (Yamaguchi et al., 2000). Recent cryo-electron microscopy analysis of spinach chloroplast 70S ribosome also demonstrated the existence of PRPS6 as a component of the 30S ribosomal subunit (Bieri et al., 2017). The amino acid sequence of PRPS6 in spinach is 82% and 22% similar to that of At1g64510 and RFC3, respectively. Although the RFC3 plastid-localization signal is functional (Horiguchi et al., 2003), and the possibility that RFC3 also functions as a plastid ribosomal protein cannot be formally discarded, low amino acid sequence conservation between PRPS6 and RFC3 has rendered the function of RFC3 in plastids obscure.

A notable feature of the LR phenotype in *rfc3* is its sucrose sensitivity; *rfc3* forms abnormal LRs when grown in media containing 3% sucrose but not in media containing 0.5% sucrose (Horiguchi et al., 2003). Sucrose concentration shift experiments showed that primary roots grown under the 3% sucrose condition are no longer able to form normal LRs even when LRs are initiated after transfer to the 0.5% sucrose condition (Horiguchi et al., 2003). This suggests that the cause of defective LR development in *rfc3* is associated with primary root growth but not the process of LR formation itself. Here, we suggest a role of plastid translation in stem cell patterning in lateral root primordia by showing that *rfc3* is defective in the accumulation of plastid rRNAs, and examining the effects of mutants defective in plastid ribosome biosynthesis and plastid translation inhibitors.

## RESULTS

### The phylogenetic origin of RFC3 is different from PRPS6

To better understand the relationship between RFC3 and the bRPS6 family proteins, we performed an extensive phylogenetic analysis. We identified the bRPS6 family proteins from land plant, green algal, red algal, animal and bacterial species (Table S1). The phylogenetic analysis revealed that the land plant sequences grouped into three different clades, such as the RFC3 clade, a clade that includes At1g64510, and a clade including At3g18760, a newly identified bRPS6 domain-containing protein from Arabidopsis (Fig. S1A). The plastid RPS6 (PRPS6) clade, which included At1g64510, *Chlamydomonas reinhardtii* PRPS6, and homologous genes of spinach PRPS6 (Yamaguchi et al., 2000, 2002; Tiller and Bock, 2014), was associated weakly with the Eurhodophytina and cyanobacterial RPS6s [bootstrap (BS) values, 20%]. The mitochondrial RPS6 (MRPS6) clade including At3g18760 was closely related to animal MRPS6s (BS values, 84%). In contrast, the RFC3 clade was most closely related to a clade containing both the β and γ proteobacterial RPS6s among the proteobacterial groups (BS values, 83%). The alignment shown in Fig. S1B indicates that the two α-helices (a1 and a2) and four β-sheets (b1–b4) in the bRPS6 domain are strongly conserved. The length of the amino acid sequence between b2 and b3 in land-plant RFC3s was the same as bacterial RPS6s compared with PRPS6s and MRPS6s, indicating that the bRPS6 domain of RFC3 is more similar to bRPS6s than to PRPS6s and MRPS6s.

### RFC3 localization in leaf and root cells

**Fig. 1.**
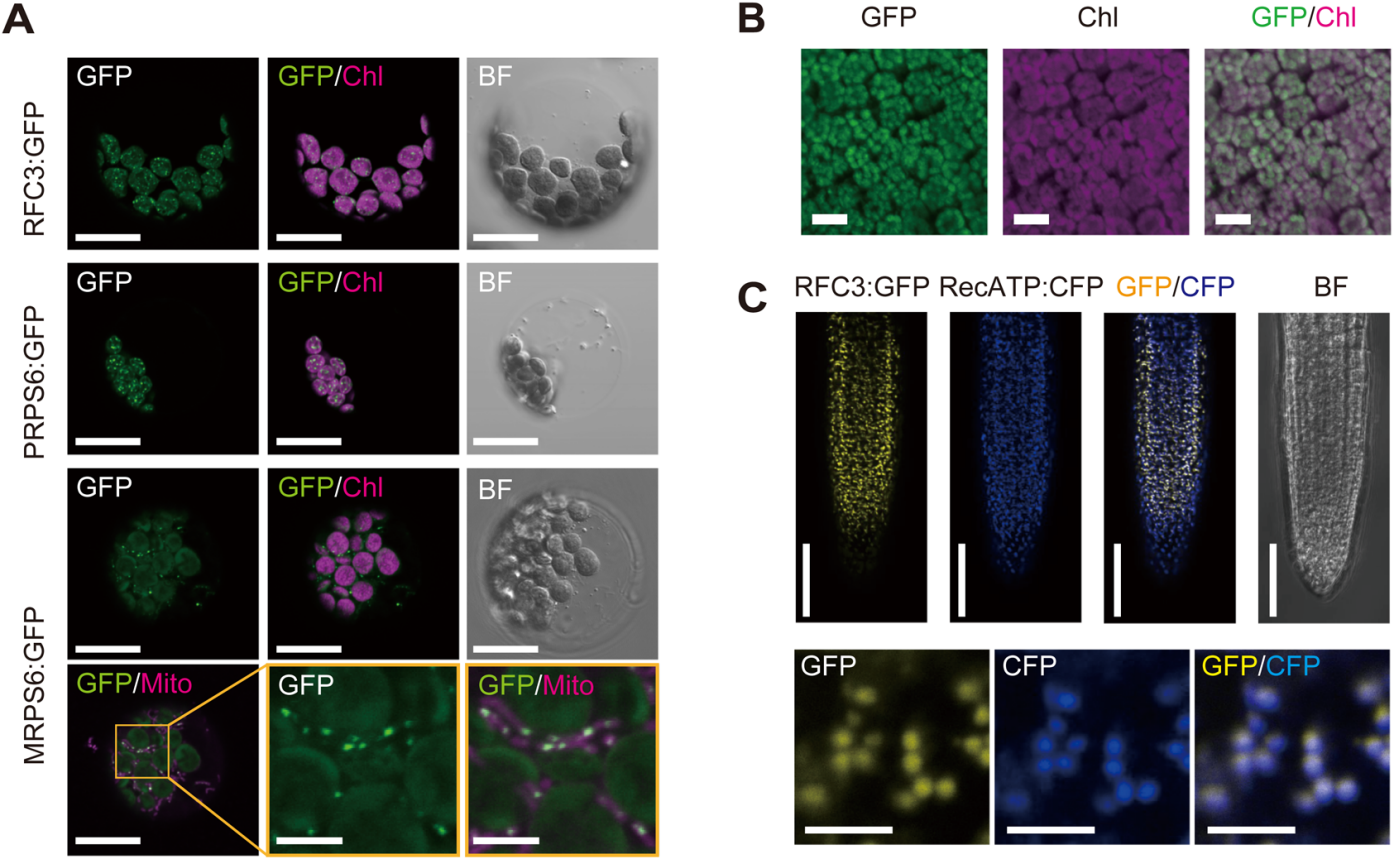
**Subcellular localization of RFC3:GFP.** (A) Transient expression assays in Arabidopsis mesophyll protoplasts. GFP signals (GFP), chlorophyll autofluorescence (Chl), the bright field (BF) image and MitoTracker Red (Mito) are shown. (B) RFC3:GFP localization in mesophyll cells of leaves of a stable *RFC3g:GFP* line. (C) Colocalization analysis of RFC3:GFP and RecA-TP:CFP in the meristematic region of lateral roots (LRs). Scale bars: 5 µm (middle and right panels in the bottom row of A, lower panels of C), 20 µm (rest of A), 50 µm (B and upper panels of C).

Transient expression analysis of *35Sp::RFC3:GREEN FLUORESCENT PROTEIN* (*GFP*), *35Sp::PRPS6:GFP*, and *35Sp::MRPS6:GFP* using mesophyll protoplasts detected GFP fluorescence from RFC3:GFP and PRPS6:GFP as a pattern of spots dispersed throughout the chloroplasts and GFP fluorescence from MRPS6:GFP predominantly as spots in the mitochondria (Fig. 1A). The same result was also observed in leaves of stable lines expressing the *RFC3* genomic fragment fused to *GFP* that complemented the root and shoot phenotypes of *rfc3* (*RFC3g:GFP*/*rfc3-2*) (Fig. 1B; Fig. S2A-C). The GFP signal in roots of *RFC3g:GFP*/*rfc3-2* plants was found in root tips and differentiated portions of primary roots as well as LR primordia (Fig. S2D,E) and detected as numerous small spots that completely overlapped with a plastid marker [cyan fluorescent protein (CFP) fused to the RECA transit peptide (*35Sp::RecA-TP:CFP*)] (Fig. 1C), demonstrating that RFC3 also localizes to plastids in roots. These results indicate that *RFC3* encodes a conserved bRPS6 protein, the origin of which is separate from those of PRPS6 and MRPS6, and which, similar to PRPS6, functions in plastids.

### Photosynthesis-related phenotypes of *rfc3*

**Fig. 2.**
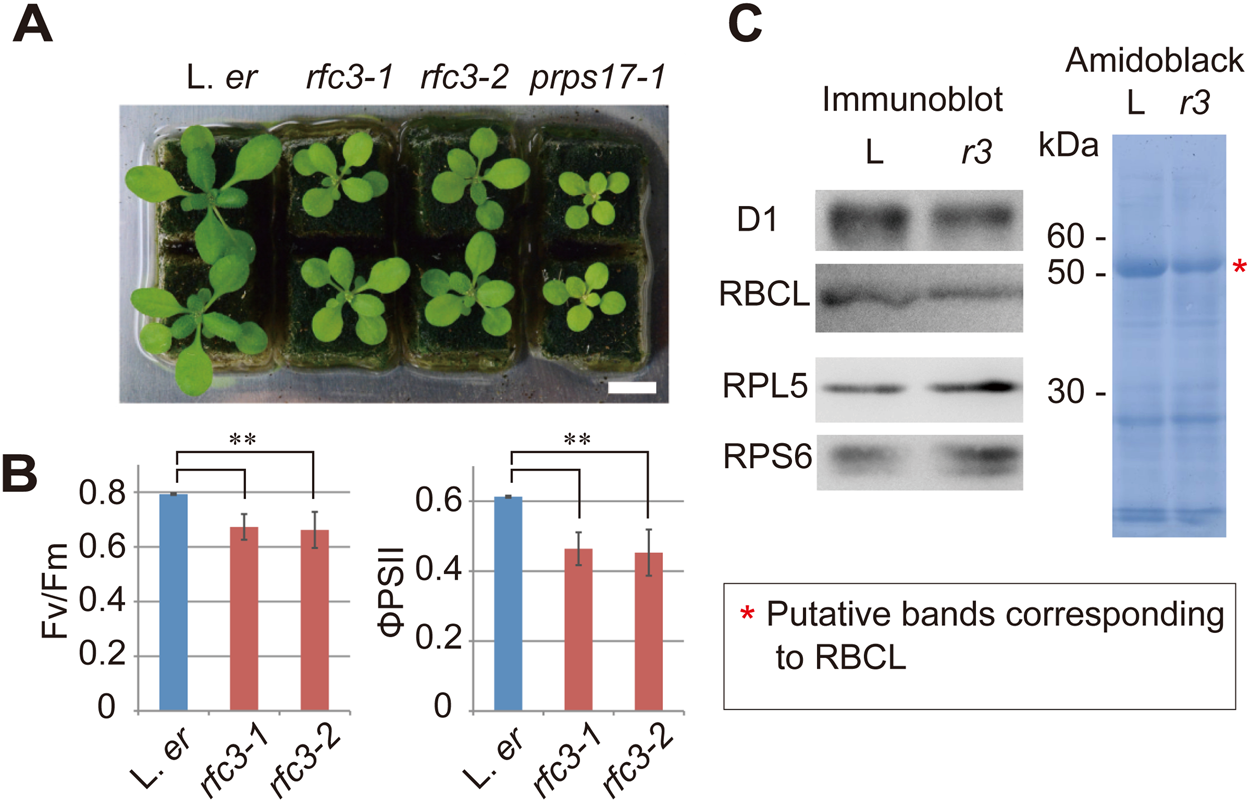
**RFC3 function in chloroplasts.** (A) Shoots from different alleles of *rfc3* and *prps17-1* mutant plants. Scale bar: 1 cm. (B) The photosynthetic parameters of L. *er* and two *rfc3* alleles [*F*v/*F*m and (*F*m′−*F*s)/*F*m′=Φ_PSII_]. The maximum fluorescence level at the closed PSII center (*F*m′) and the steady-state fluorescence level (*F*s) were determined in actinic light (120 µmol photons m^–2^ s^–1^). (C) Immunoblot analysis and Amido Black staining of proteins extracted from L. *er* (shown as ‘L’) and *rfc3-2* (shown as ‘*r3*’) shoots. Statistical analyses in B were carried out using the paired Student's *t-*test (**P<0.01).

In our previous work, we focused on LR development in *rfc3* (Horiguchi et al., 2003). To better understand the general characteristics of *rfc3*, we examined shoot and photosynthesis-related phenotypes. Both *rfc3-1* and *rfc3-2* are in the Landsberg *erecta* (L. *er*) background. These mutants have pale-green leaves and are smaller than wild type in young seedlings (Fig. 2A). However, leaves of *rfc3-2* reached a size similar to L. *er* when they were grown until flowering (Fig. S3A,B). The number of leaves produced by the time of flowering was slightly fewer in *rfc3-2* than in wild type (Fig. S2C). Compared to L. *er*, *rfc3* alleles showed a significantly decreased maximum quantum yield of photosystem II (PSII) (*Fv*/*Fm*), and significantly decreased quantum yields of PSII (Φ_PSII_) according to analysis using a pulse amplitude modulation fluorescence system (Fig. 2B). In three of the four *rfc3-1* plants and two of the four *rfc3-2* plants tested, the steady state level of fluorescence (*F*s) decreased below the *F*o level upon exposure to actinic light (120 µmol photons m^–2^ s^–1^), which was in contrast to wild-type plants (Fig. S4). Consequently, we concluded that photosynthetic electron transport was partially affected by the mutations. Additionally, two plastid-encoded proteins, D1 (encoded by *psbA*) and the large subunit of ribulose-l,5-bisphosphate carboxylase/oxygenase (RBCL), were slightly less abundant in *rfc3-2* shoots, whereas two nuclear-encoded cytosolic ribosomal proteins, RPS6 and RPL5, were slightly more abundant (Fig. 2C). A decrease in RBCL was also detected in *rfc3-2* by Amido Black staining (asterisk in Fig. 2C). These results indicate that RFC3 contributes to chloroplast function.

### Plastid gene expression in *rfc3*

**Fig. 3.**
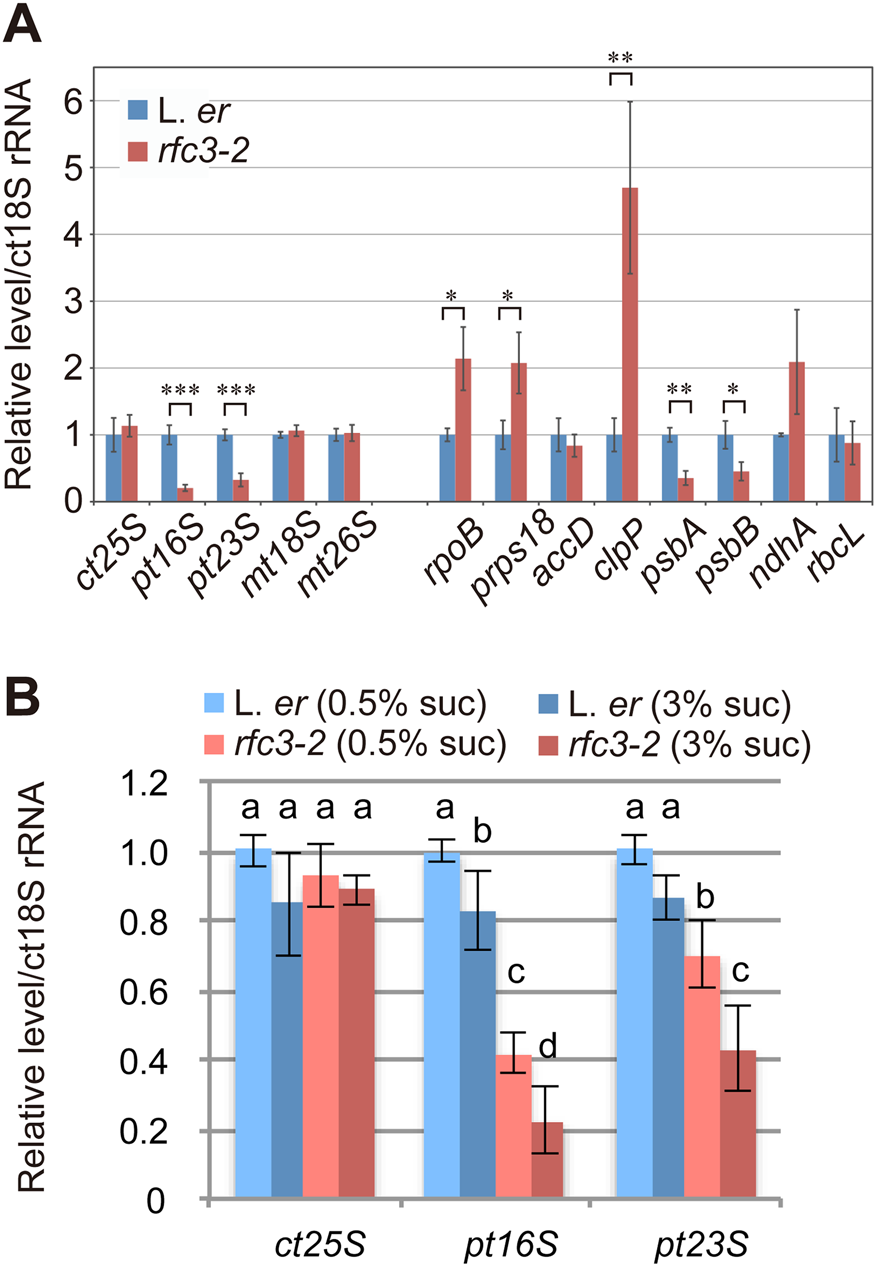
**Expression of plastid encoded genes in L. *er* and *rfc3-2* roots.** (A) Relative RNA levels in roots estimated by RT-qPCR analysis. Plants were grown in media containing 3% sucrose. (B) Effects of sucrose on the levels of plastid 16S (pt16S) and pt23S rRNAs in wild-type and *rfc3-2* roots grown in media containing 0.5% or 3% sucrose. Data are mean±s.d. (*n*=3). Statistical analyses in A were carried out using the paired Student's *t-*test (*P<0.05, **P<0.01, ***P<0.001); those in B were carried out using two-way ANOVA with Tukey HSD test (*P*<0.05). Data that are not significantly different are labelled by the same letter. In A and B, ct25S, mt18S and mt28S indicate cytosolic 25S rRNA and mitochondrial 18S and 28S rRNAs.

To test whether RFC3 has an active role in root plastid function, we investigated the effect of the *rfc3* mutation on the expression of plastid-encoded genes in roots grown in media containing 3% sucrose (Fig. 3A). Compared with cytosolic 18S rRNA (ct18S), the expression of plastid 16S (pt16S) and pt23S rRNAs was dramatically reduced, suggesting a corresponding decrease in plastid ribosomes. In addition, *psbA* and *psbB* decreased significantly in *rfc3-2* roots. In contrast, expression of *rpoB*, *prps18* and *clpP* increased significantly in *rfc3-2*. However, the expression of plastid-encoded *accD*, *ndhA* and *rbcL*, and the expression of ct25S, mitochondrial 18S (mt18S) and mt26S rRNAs did not differ significantly between wild type and *rfc3-2*.

Because abnormal LRs do not form in *rfc3* grown in media containing 0.5% sucrose (Horiguchi et al., 2003), we examined whether plastid rRNA levels are also affected by sucrose conditions. Hereafter, we designate sucrose conditions as follows: 0% sucrose=Suc0, 0.5%=Suc0.5, and 3%=suc3. Compared to wild type, expression levels of both pt16S and pt23S rRNAs in the *rfc3-2* roots decreased by ∼60% and 30%, respectively, even when they were grown in Suc0.5 media. These rRNA levels further decreased in Suc3 media (Fig. 3B). In *rfc3-2* roots, the level of pt16S rRNA showed a greater decrease than pt23S rRNA in both Suc0.5 and Suc3 media (Fig. 3B). These results suggest that a high concentration of sucrose enhances a negative effect of the *rfc3* mutation on LR development through decreases in plastid rRNA levels.

### *rfc3* mutations result in abnormal stem-cell patterning in LRs

**Fig. 4.**
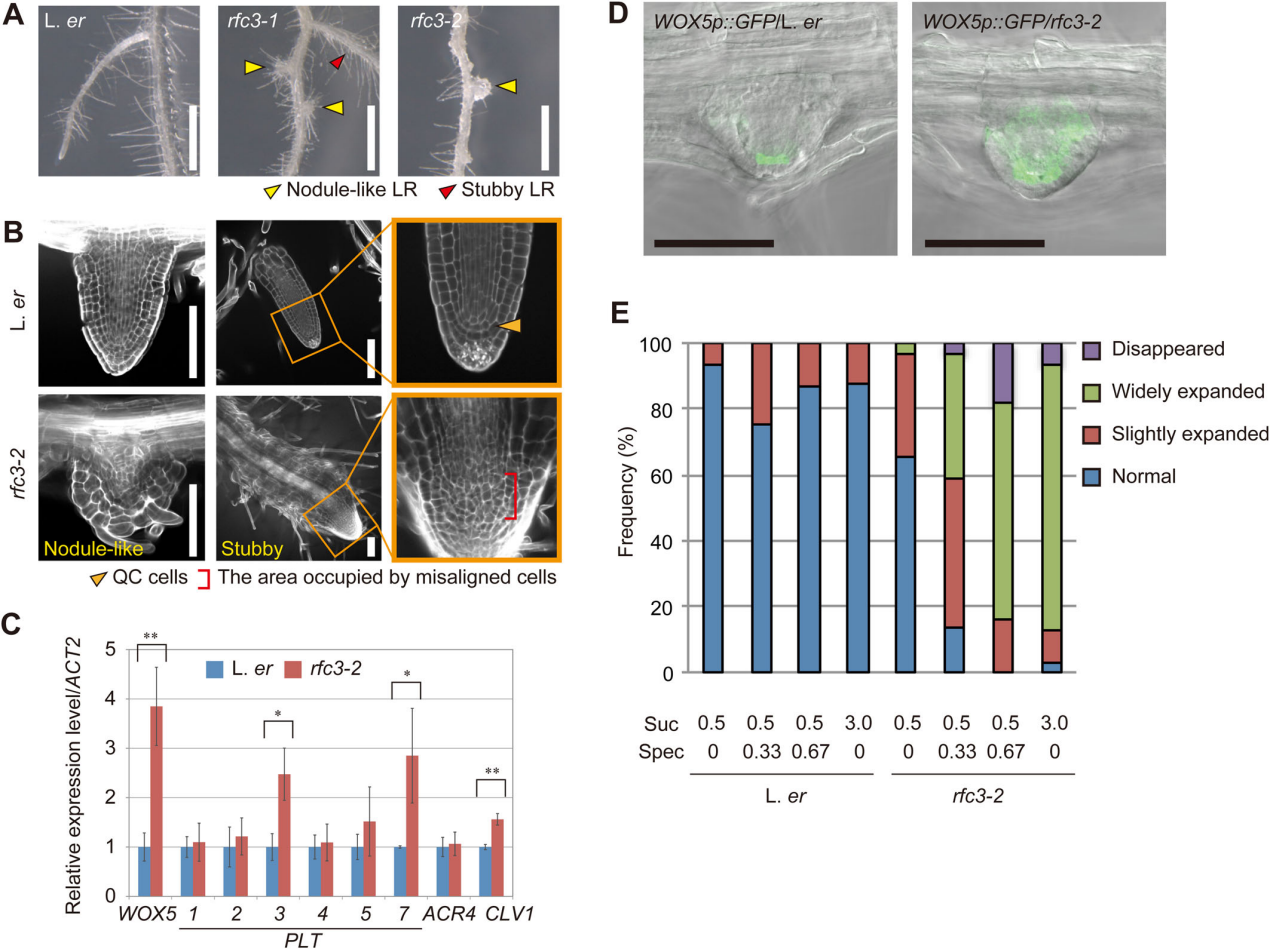
**Characterization of the *rfc3* LR phenotype.** (A) The LR phenotype of *rfc3* alleles. Yellow arrowheads indicate nodule-like LRs; red arrowhead indicates a stubby LR. (B) Nodule-like LRs (left panel) and stubby LRs (middle and right panels) observed by modified pseudo-Schiff-propidium iodide (mPS-PI) staining. Orange arrowhead indicates QC cells, red bracket indicates the area occupied by misaligned or stratified cells. (C) Relative expression of root stem cell regulatory genes in roots measured by RT-qPCR (*n*=3). Statistical analyses were carried out as described in Fig. 3. (D) *WOX5p::GFP* patterns (green) merged with bright field images in LR primordia. (E) Phenotype frequencies of *WOX5p:GFP* in L. *er* and *rfc3-2* LRs grown in different concentrations of sucrose and Spec. Sucrose and Spec concentrations are indicated by percentage and mg l^−1^, respectively. Individual LR primordia were classified into normal, slightly expanded, widely expanded and disappeared *WOX5p::GFP* expression patterns. LR primordia beyond stage VI and LRs up to 200 µm were scored. The total number of LR primordia plus LRs in each condition was >76. Scale bars: 1 mm (A), 100 µm (B,D). Statistical analyses were carried out as described in Fig. 3.

*rfc3-1* and *rfc3-2* grown on the Suc3 media form nodule-like LRs (Fig. 4A, yellow arrowheads) (Horiguchi et al., 2003), which are round or pointed and covered with epidermal and root hair cells. They were likely to lose distal tissues and the quiescent center (QC) cells (Fig. 4B) (Horiguchi et al., 2003). In this study, we noticed that *rfc3-2* also formed stubby LRs, which occurred at a lower frequency than the nodule-like LRs (Fig. 4A, red arrowhead). The stubby LRs were shorter and thicker than the wild-type LRs, but had tissue resembling a root cap at the apex. A group of cells located between proximal and distal tissues in stubby LRs of *rfc3* was disorganized or multi-layered, indicating that the stem-cell alignment collapsed (Fig. 4B). We previously showed that *rfc3* forms nodule-like LRs when grown on the Suc3 media but it is able to develop normal LRs on the Suc0.5 media (Horiguchi et al., 2003). To examine if the observed stubby LRs are a milder LR phenotype, we cultured *rfc3* in media containing different concentrations of sucrose (Fig. S5A). Wild-type plants grown in all sucrose conditions examined and *rfc3-2* grown in Suc0 media formed neither stubby nor nodule-like LRs. In *rfc3-2,* stubby and nodule-like LRs began to form in Suc0.5 media, although their frequencies were less than 5%. As expected, the frequency of stubby LRs was higher in intermediate sucrose concentrations than in lower or higher sucrose conditions. The frequency of nodule-like LRs steadily increased as the sucrose concentration increased. These results suggest that stubby LR is a less severe form of the *rfc3* LR phenotype. On the other hand, the length of primary roots in *rfc3-2* was always shorter than wild type in any sucrose condition tested (Fig. S5B). The growth defect of the *rfc3-2* primary root was slightly enhanced in the presence of sucrose, as indicated by the relative primary root length of *rfc3-2* compared to wild type (Fig. S5B).

We also examined whether sucrose affected LR development as an osmoticum or metabolizable sugar (Fig. S5C). Three percent sucrose corresponds to 88 mM, and we examined the effect of glucose as another metabolizable sugar and mannitol as a non-metabolizable osmoticum at the same molar concentration. Growth under no metabolizable sugars often causes vitrification. To avoid this, 0.5% sucrose was added to media containing glucose or mannitol. In the presence of glucose but not mannitol, *rfc3* formed shorter primary roots and abnormal LRs (Fig. S5C), suggesting that the presence of metabolizable sugars is a trigger of abnormal LR development.

Irregular organization of LR cells motivated us to examine the expression of a number of well-characterized root stem-cell regulatory genes, including *WUSCHEL-RELATED HOMEOBOX5* (*WOX5*) (Sarkar et al., 2007), *PLETHORA3* (*PLT3*), *PLT7* (Galinha et al., 2007), *CLAVATA1* (*CLV1*) (Stahl et al., 2013) and *ARABIDOPSIS CRINKLY4* (*ACR4*) (De Smet et al., 2008) (Fig. 4C). The expression levels of *WOX5*, *PLT3*, *PLT7*, *CLV1* and *ACR4* were higher in roots of *rfc3-2* grown in Suc3 media. Expression of *WOX5p::GFP* was limited to the QC in LR primordia of the wild type but was more widespread in almost all LR primordia of *rfc3-2* grown in Suc3 media (Fig. 4D,E). Conversely, GFP fluorescence was not found in ∼5% of *rfc3-2* LR primordia (Fig. 4E). These LRs formed root hairs on their apical parts, suggesting that cells in these LRs were fully differentiated. These results indicate that *RFC3* is required for normal LR development and for the proper expression of a subset of root stem cell regulatory genes.

Experiments with Suc3 media may not reflect natural growth. To minimize the effect of exogenous sucrose, we used Suc0.5 media and checked expression patterns of *WOX5p::GFP* (Fig. 4E; Fig. S6). Although *rfc3-2* grown on Suc0.5 media had only a few nodule-like LRs, we found a slight expansion of GFP signal in the LR apex of ∼35% of *rfc3-2* plants (Fig. 4E; Fig. S6). These results, together with a finding that *rfc3* grown in Suc0.5 media had less plastid rRNAs (Fig. 3), suggest that RFC3 is required to accumulate plastid rRNAs to a sufficiently high level to ensure normal LR development under a near-physiological condition. Suc3 further decreased plastid rRNA levels in *rfc3-2* and this is very likely a cause of sugar-sensitive LR defects.

### Spectinomycin (Spec)-treated L. *er* plants phenocopy the *rfc3* root defects

**Fig. 5.**
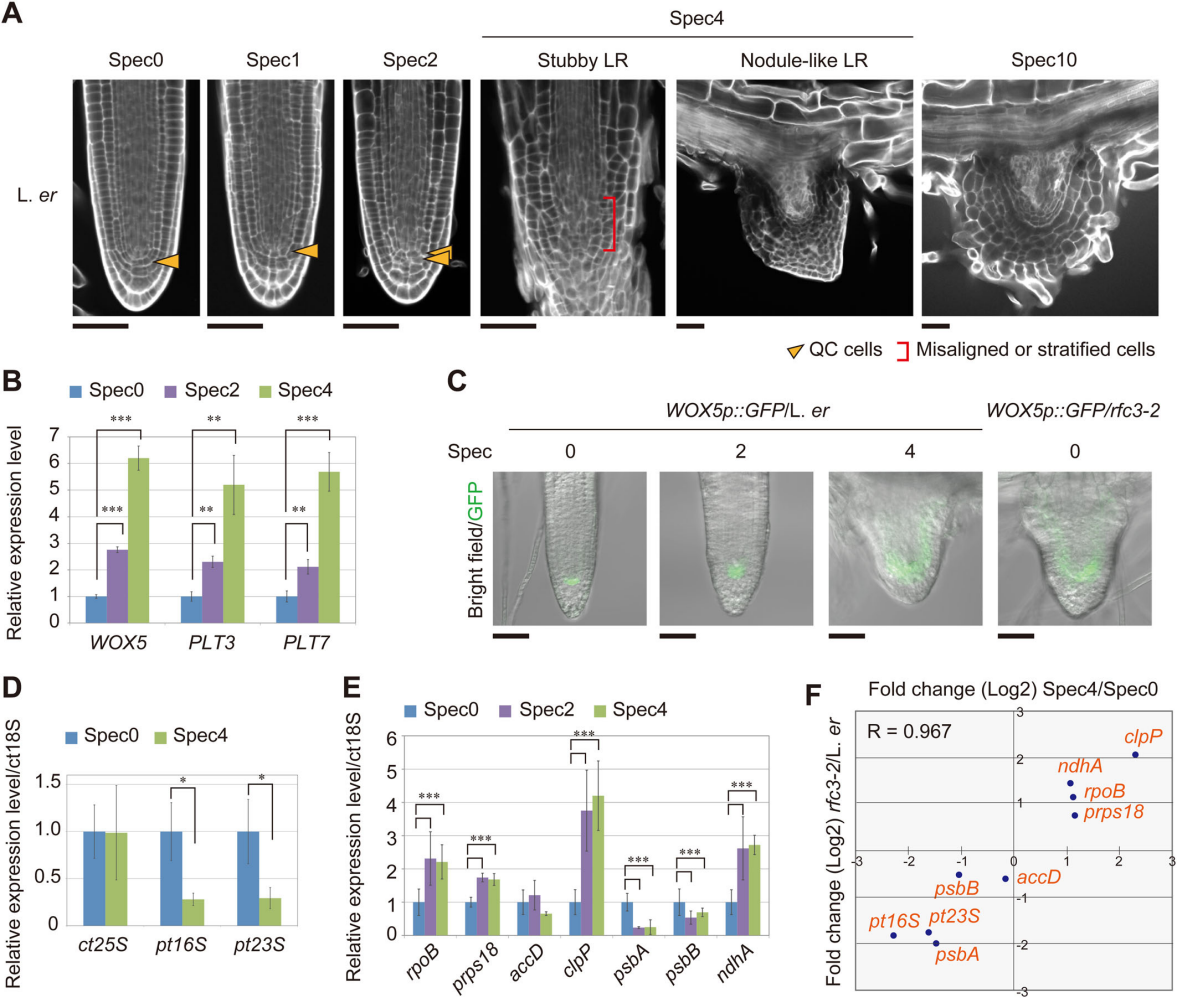
**Effect of the plastid translation inhibitor Spec on LR development.** (A) mPS-PI-stained LRs of L. *er* plants grown on medium containing various Spec concentrations. Arrowheads indicate the position of the QC cells, brackets indicate the area occupied by misaligned or stratified cells. (B) Relative expression levels of *WOX5*, *PLT3*, and *PLT7* in Spec-treated plants (*n*=3). (C) Distribution of GFP fluorescence (green) in *WOX5p::GFP* merged with bright field images. (D,E) Relative transcript levels of plastid ribosomal RNAs (D) and plastid-encoded genes (E) in roots (*n*=3). (F) Correlation analysis of changes in expression (Log_2_) in plastid-encoded genes resulting from the *rfc3* mutation and 4 mg l^−1^ Spec treatment. R, Pearson's correlation value. Statistical analyses were carried out as described in Fig. 3. Scale bars: 50 µm (A,C).

Our findings suggest that the *rfc3* mutation impaired plastid gene expression, resulting in abnormal LR stem-cell patterning. Because RFC3 is a bRPS6 family protein and its mutation is associated with a reduction in pt16S and pt23S rRNAs, impaired translation may have effects similar to those observed in *rfc3*. Therefore, we investigated the effect on LR development of three bacterial ribosome inhibitors, Spec, kanamycin and streptomycin, which inhibit plastid translation (Fromm et al., 1987; Svab and Maliga, 1991; Kavanagh et al., 1994; Rosellini et al., 2004; Conte et al., 2009; Parker et al., 2014) in Suc3 media. We observed that plants treated with 4 and 10 mg l^−1^ Spec (designated Spec4 and Spec10, respectively, and so forth) in Suc3 media formed nodule-like and/or stubby LRs with misaligned stem cells that resembled those of *rfc3* (Fig. 5A; Fig. S7). Treatment with Spec2, but not Spec1, produced slight irregularities in LR stem-cell patterning without generating macroscopic morphological changes (Fig. 5A). Increasing the Spec concentration from Spec0 to Spec2 resulted in gradual increases in the expression of *WOX5*, *PLT3* and *PLT7* and more widespread expression of *WOX5p::GFP* (Fig. 5B,C). In addition, the kanamycin and streptomycin treatments induced nodule-like and/or stubby LR formation in L. *er* plants (Fig. S7).

We also examined the effect of Spec2 and Spec4 on L. *er* plants and found that plastid rRNA levels and *psbA* and *psbB* transcripts were downregulated (Fig. 5D,E), whereas the transcript levels of plastid-encoded genes *rpoB*, *prps18*, *clpP* and *ndhA* were upregulated. A good correlation was observed between the RNA levels of plastid-encoded genes in *rfc3* and Spec4-treated wild-type plants (Fig. 5F).

In general, drug treatment at a high concentration may cause non-specific deleterious effects on development. To further confirm the relationship between plastid translation and abnormal LR development, we analyzed the effects of Spec treatment on the *WOX5p::GFP* expression pattern in *rfc3* LRs formed in Suc0.5 media (Fig. 4E). We first examined wild type, and found that Suc0.5 combined with Spec0.33 or Spec0.67 did not affect the *WOX5p::GFP* expression pattern (Fig. 4E; Fig. S6). On the other hand, *WOX5p::GFP* expression in *rfc3-2* LR primordia was expanded or disappeared even in Spec0.33 media (Fig. 4E; Fig. S6). Most of the LRs with altered *WOX5p::GFP* expression were stubby or nodule-like ones (Fig. S6). Abnormal LRs induced at a low concentration of Spec suggest that this phenotype results from the inhibitory effects on plastid translation rather than non-specific effects.

### Plastid translation-deficient mutants trigger an *rfc3*-like LR phenotype with a low concentration of Spec

**Fig. 6.**
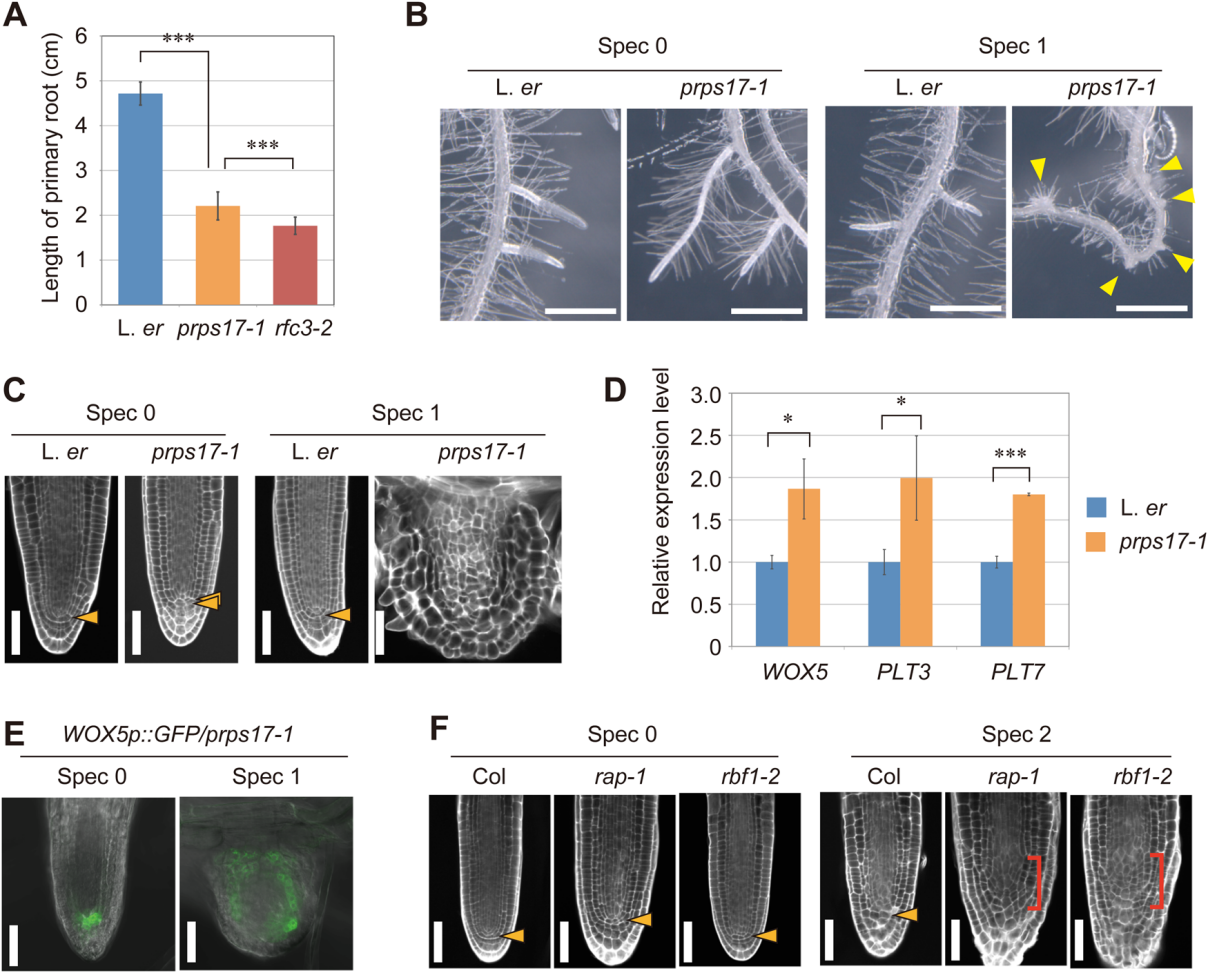
**LR phenotype of plastid translation-defective mutants with/without Spec.** (A) Primary root length of L. *er*, *prps17-1* and *rfc3-2* at 8 days post-sowing. Data are mean±s.d. (*n*≥46). ***P<0.001 by Tukey's HSD test. (B) LR phenotype of L. *er* and *prps17-1* with or without Spec. Yellow arrowheads indicate nodule-like LRs. (C) mPS-PI-stained LR phenotype of L. *er* and *prps17-1* with or without Spec. Orange arrowheads indicate the position of the QC cells. (D) Relative expression levels of *WOX5*, *PLT3* and *PLT7* in L. *er* and *prps17-1* roots without Spec. Data are mean±s.d. Experiments were performed in biological triplicate. ***P<0.001 and *P<0.05 by paired Student's *t-*test. (E) The GFP signal (green) of *WOX5p::GFP* in *prps17-1* background with or without Spec. The differential interference contrast images were merged. (F) mPS-PI-stained LR phenotype in *rap-1* and *rbf1-2* with or without Spec. Orange arrowheads indicate the position of the QC cells, red brackets indicate the area occupied by disorganized or stratified cells. Scale bars: 1 mm (B), 50 µm (C,E,F).

The similar effect of *rfc3* mutation with Spec and other drugs supports the idea that abnormality in LR stem cell patterns is due to impaired plastid translation. For further confirmation, we examined plastid translation-deficient mutants. First, we studied the phenotype of *prps17-1*, which is an L. *er* background mutant deficient in a nuclear-encoded plastid ribosomal protein (Romani et al., 2012). The shoot size of *prps17-1* was smaller than wild type and *rfc3-2* (Fig. 2A). On the other hand, primary root growth of *prps17-1* was suppressed but the severity was milder than *rfc3-2* (Fig. 6A). Growth of LRs in *prps17-1* was not slowed (Fig. 6B), but showed a slightly irregular stem-cell alignment (Fig. 6C) with a slightly expanded GFP fluorescence pattern of *WOX5p::GFP* (Fig. 6E), and the expression of *WOX5*, *PLT3*, and *PLT7* increased slightly (Fig. 6D). Then, we applied a low concentration of Spec to *prps17-1*. Unlike Spec1-treated L. *er* plants, Spec1-treated *prps17-1* plants formed nodule-like LRs (Fig. 6C) with a widely distributed GFP signal of *WOX5p::GFP* (Fig. 6E) that resembled those of untreated *rfc3* mutants (Fig. 4D).

As described above, *prps17-1* showed the same effect as *rfc3-2* in low concentration Spec treatments. To examine the genetic interaction between these two mutations, we attempted to create a *prps17-1 rfc3-2* double knockout mutant. However, we were unable to find any *prps17-1 rfc3-2* double homozygous mutants in the F2 population. *prps17-1 rfc3-2*/+ plants were selected and the genotype of their descendants was examined. Among the 56 plants, 23 and 33 plants had the *prps17-1* and *prps17-1 rfc3-2*/+ genotypes, respectively, but no *prps17-1 rfc3-2* double mutant was found. Seed development and maturation in fruits of the *prps17-1* single mutant and *prps17-1 rfc3-2*/+ double mutant were observed (Fig. S8A). All seeds of *prps17-1* fruits grew green during seed maturation (*n*=91), while 21.6% of *prps17-1 rfc3-2*/+ fruit seeds remained albino (Fig. S8A, central panels, *n*=218). We did not detect albino seeds from the fruits of L. *er* and *rfc3-2* (*n*=134 and 120, respectively). In the *prps17-1 rfc3-2*/+ fruits, the green seeds were in the mature green stage and the embryos were spherical (Fig. S8A,B) but those in albino seeds were still in a globular stage (Fig. S8C,D). In the late stage of *prps17-1 rfc3-2*/+ fruit, there was no albino seed, but brown shrinking seeds were found (Fig. S8A, lower panels). Shrinking seeds were not observed in *prps17-1* fruit. It is assumed that albino/shrinking seeds are *prps17-1 rfc3-2* double mutants, and both mutations work synergistically, resulting in the arrest of embryonic development during the globular phase. Similar to this observation, mutations in several nuclear-encoded plastid ribosomal protein genes result in an embryonic lethal phenotype (Romani et al., 2012). The synergistic effects of the *rfc3* and *prps17* mutations suggest that the function of RFC3 is closely related to the function of PRPS17, most likely translation or plastid ribosome biogenesis, during development.

**Fig. 7.**
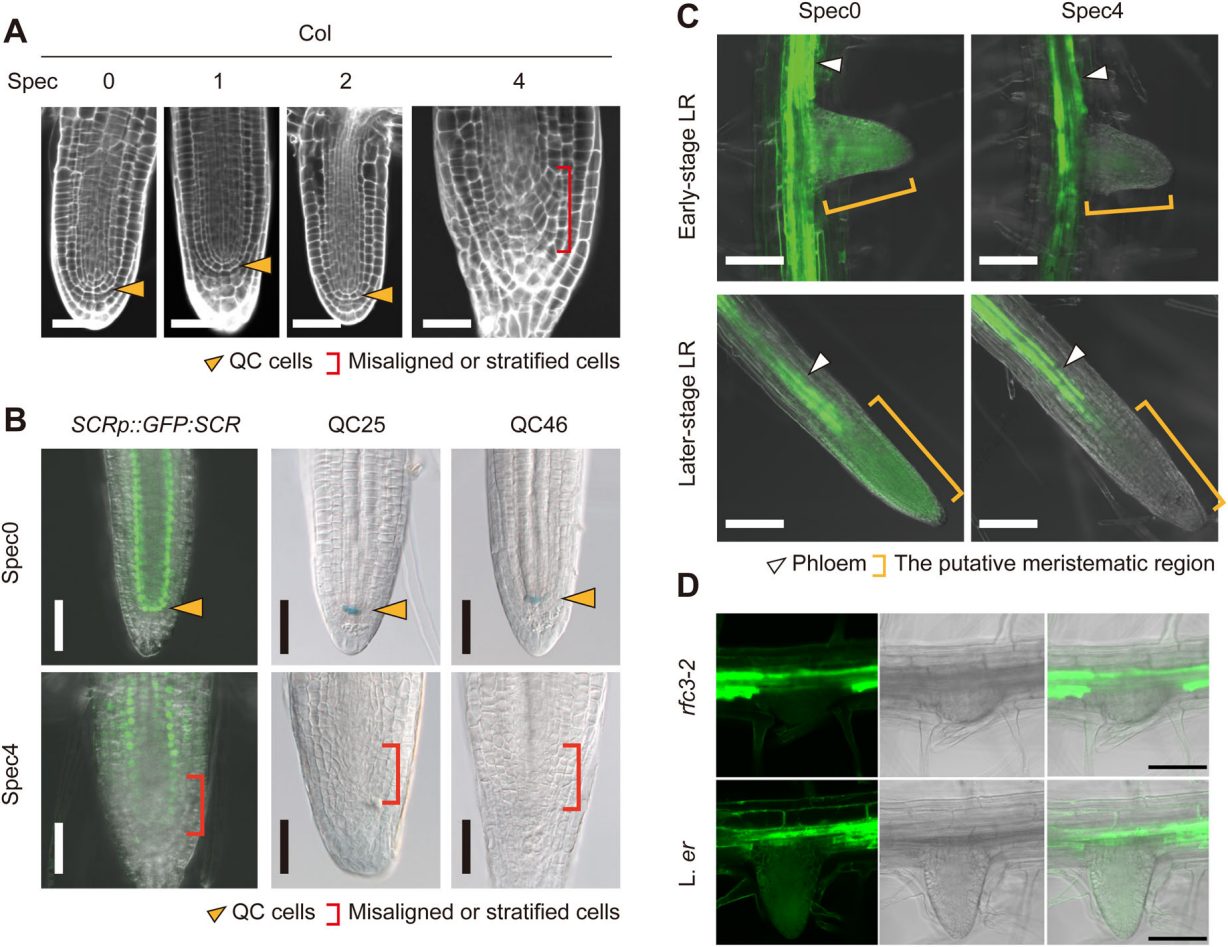
**Spec treatment of Col-0-background meristem marker lines and the *SUC2p::sGFP* line.** (A) mPS-PI-stained LRs of Col-0 plants treated with Spec. (B) *SCRp::GFP:SCR/scr*, QC25, and QC46 expression patterns. Orange arrowheads indicate the position of the QC cells, red brackets indicate the area occupied by misaligned or stratified cells. (C,D) GFP patterns in LRs of Spec4-treated *SUC2p::sGFP* in the wild-type Col-0 (C) and *rfc3-2* (D) backgrounds. The green signals in B, C and D are GFP fluorescence; the blue signal in B is β-glucuronidase (GUS)-stained cells. White arrowheads indicate phloem, orange brackets indicate the meristem region. Scale bars: 50 µm (A,B), 100 µm (C,D).

Next, we examined Col-0-background plastid translation-deficient mutants, *rap-1* and *rbf1-2*. RAP and RBF1 are involved in the maturation of pt16S rRNA (Fristedt et al., 2014; Kleinknecht et al., 2014). LR phenotypes of *rap-1* and *rbf1-2* were indistinguishable from those of wild-type Col-0 (Fig. 6F). Then, we analyzed the Spec sensitivity of wild-type Col-0 and their mutants. Spec4-treated Col-0 plants formed *rfc3*-like stubby LRs with abnormal stem cell alignment (Fig. 7A). Spec1-treated Col-0 LR did not differ from untreated Col-0 (Fig. 7A), but Col-0 plants treated with Spec2 showed variable LR phenotypes: 85.2% of the plants showed a wild-type-like phenotype but the others (14.8%) showed weak stratification of stem cells (*n*=27; Figs 6F and 7A). On the other hand, all of the Spec2-treated *rap-1* mutants formed nodule-like LRs and/or stubby LRs with a severe defect in stem cells (*n*=18) and most Spec2-treated *rbf1-2* mutants had abnormal LRs with a weak or severe stem cell defect (88.9%, *n*=27; Fig. 6F).

The synergistic effects of plastid translation-deficient mutants, *prps17-1*, *rap-1* and *rbf1-2*, and Spec treatment on the LR phenotype support the hypothesis that impaired plastid translation and/or plastid ribosome biogenesis triggers abnormal LR development.

### Spec treatment alters characteristics of LR meristematic cells

As described above, Spec4 treatment results in LR deformation. We then investigated the effect of Spec4 treatment on root meristem markers. The GFP signal from a *SCARECROW* (*SCR*) reporter line (*SCRp::GFP:SCR/scr-3*) was detectable in both the endodermis and the QC in untreated plants, but was faint in the meristematic region of stubby LRs in Spec4-treated plants (Fig. 7B). Additionally, no signals were detected from the QC-specific markers QC25 and QC46 in Spec4-treated plants (Fig. 7B). We also found that QC25 expression in stubby and nodule-like LRs of *rfc3-2* was absent while its expression was maintained in the primary root tip (*n*=10, Fig. S9). These results, together with the altered expression of *WOX5p::GFP* (Fig. 5C), indicate that the stubby LRs formed in Spec-treated wild-type plants also have abnormal meristematic characteristics.

The *SUCROSE-PROTON SYMPORTER2p* (*SUC2p*)*::sGFP* line can be used to visualize symplasmic connectivity between the phloem and the meristematic regions in roots by monitoring cytosolic GFP (Imlau et al., 1999). GFP fluorescence was detected in the meristematic region of LRs in untreated *SUC2p::sGFP* plants, whereas it was detected only in the phloem of LRs in Spec4-treated *SUC2p::sGFP* plants (Fig. 7C). *SUC2p::sGFP* in *rfc3-2* also showed restricted diffusion of GFP into LR primordia (Fig. 7D). These results show that the symplastic connectivity between phloem tissues and LR primordia is controlled by the common pathway affected by the Spec treatment and the *rfc3* mutation.

### The intracellular distribution of plastids was altered in *rfc3*

**Fig. 8.**
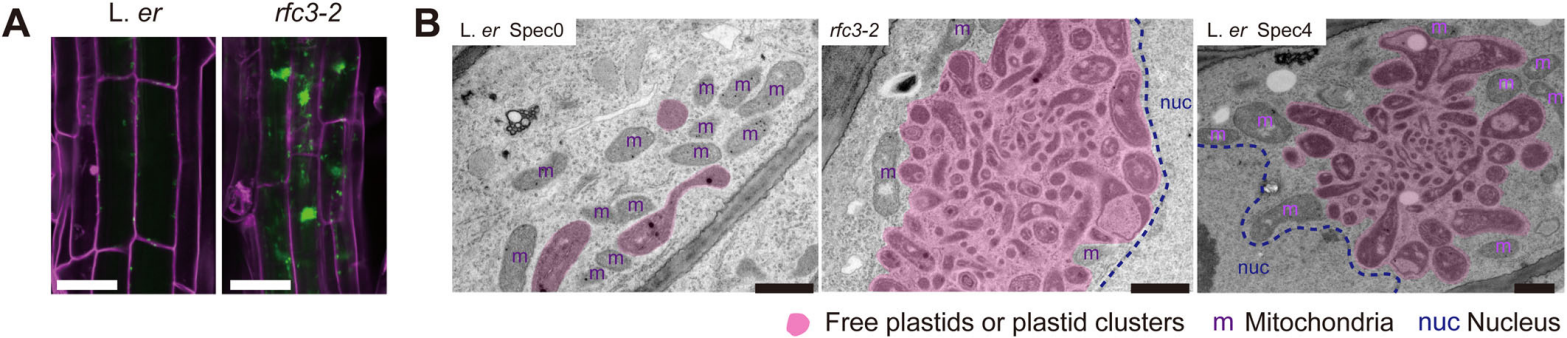
**Plastid distribution in root cells.** (A) Intracellular distribution of CFP in the *35Sp::RecA-TP:CFP* lines in L. *er* and *rfc3-2* backgrounds. CFP fluorescence is shown in green and roots were stained with PI (magenta). (B) Ultrastructure of plastids in pericycle cells of primary roots in L. *er*, *rfc3-2*, and Spec4-treated L. *er*. Scale bars, 50 µm (A), 1 µm (B).

*RAP* encodes an octotricopeptide repeat protein localized in chloroplasts and its loss of function results in a pt16S rRNA maturation defect (Kleinknecht et al., 2014). Interestingly, an earlier report of *rap* described its enhanced resistance to pathogen infection (Katiyar-Agarwal et al., 2007). A recent study also highlighted the involvement of chloroplasts in an immune response during which chloroplasts elongate tubular structures known as stromules. These stromules make contact with the nucleus and this dynamic behavior of chloroplasts is proposed to have a signaling role (Caplan et al., 2015). Curiously, we also found that *rfc3-2* and Spec-treated wild type shared abnormalities in behavior of root plastids. In experiments using *35Sp::RecA-TP:CFP*, the CFP fluorescence in wild-type plants was observed as small spots scattered throughout the root cells (Fig. 8A). Conversely, the CFP fluorescence in *rfc3-2* mutants and Spec4-treated plants was visible as abnormal aggregations in the center of cells and small spots in the mature parts of the primary root (Fig. 8A; Fig. S10). Similar aggregation was observed in Spec4-treated *RFC3g:GFP*/*rfc3-2* (Fig. S10). Transmission electron microscopy observations revealed that plastids in the pericycle cells of mature untreated L. *er* primary root were dispersed, whereas those of *rfc3* primary roots often formed large plastid clusters that contained mitochondria and other structures (Fig. 8B; Fig. S11). The cluster of plastids was also observed in cells of Spec-treated wild-type primary roots (Fig. 8B; Fig. S11). These findings demonstrate that impaired translation in plastids affects intracellular distribution of root plastids.

## DISCUSSION

We reported the *RFC3* gene, which was cloned nearly 15 years ago as a member of PRPS6 but with a very low similarity compared to authentic PRPS6 in Arabidopsis and spinach (Horiguchi et al., 2003). In this study, detailed phylogenetic analyses supported that RFC3 and its orthologs are present only in land plants and are likely of β/γ-proteobacterial origin (Fig. S1). We found that *rfc3* reduces plastid rRNAs in roots (Fig. 3), suggesting that RFC3, as a non-ribosomal protein, plays a role either during ribosome biogenesis or in stabilizing mature ribosomes.

In this study, we also demonstrated that *rfc3* grown in Suc3 media, Spec-treated wild-type plants, and plastid ribosome-related mutants commonly produced abnormal LRs that lacked typical stem-cell patterning (Figs 4-6). These findings suggest a link between impaired translation and/or ribosome biogenesis in plastids and abnormal LR development. However, there are two critical issues concerning the *rfc3* phenotype and Spec treatment. For *rfc3*, its sucrose-sensitive abnormal LR formation raises a question about whether the observed phenotype is physiologically relevant. We found that stubby and nodule-like LRs begin to form at a low frequency in a media containing sucrose as low as 0.5% and the frequency of their appearance increased as sucrose concentration increased (Fig. S5). Thus, *rfc3-2* grown in a low sucrose condition is already vulnerable to impaired plastid translation. Further reduction in plastid rRNA levels by sucrose (Fig. 3) is a likely cause of sucrose-sensitive abnormal LR development. For Spec-treatment, nodule-like LRs were induced at a concentration higher than 4 mg l^−1^, which might have resulted from non-specific deleterious effects (Fig. 5). However, similar phenotypes were found when wild-type plants were treated with plastid translation inhibitors kanamycin and streptomycin, which are structurally unrelated to Spec (Fig. S7). In addition, *rfc3* grown in Suc0.5 media formed abnormal LRs at an increased frequency when they were treated with Spec0.33 while wild-type plants developed normal LRs (Fig. 4; Fig. S6). Taking these results together, we propose that *rfc3* and Spec-treatment induce abnormal LRs specifically through their negative effects on ribosome biogenesis or translation in plastids.

Our finding that exogenous sucrose has an effect to further reduce plastid rRNA levels in *rfc3* (Fig. 3B) raises a next question concerning the action of sucrose. We ruled out the possibility that osmotic stress induced by exogenous sugars is the cause of the *rfc3* phenotypes since sucrose and glucose but not mannitol induced abnormal LR formation in *rfc3* (Fig. S5C). Sucrose is metabolized into glucose and both sugars act as energy source. At the same time, glucose has a signaling role by which diverse processes from gene expression, metabolism, and development are regulated (Sheen, 2014). Whether the sucrose-dependent *rfc3* phenotypes were induced via a glucose signaling network would be examined by experiments on the relationship between ribosomal rRNA accumulation and glucose signaling regulators, such as HEXOKINASE1, SNF-related protein kinases KIN10/11 and TARGET OF RAPAMYCIN. Alternatively, plastid rRNA levels may be influenced in an indirect manner. Sucrose activates growth and cytosolic ribosome biogenesis through transcriptional induction of ribosome biogenesis factors (Kojima et al., 2007; Maekawa et al., 2017). Since plastids are one of major sites of the primary metabolism, demand of plastid gene expression machineries would be increased in response to exogenous sucrose to support growth stimulation. If cytosolic and plastid ribosomes are coordinately increased, the relative levels among cytosolic and plastid rRNAs will be unaltered significantly. The rRNA levels shown in Fig. 3 were expressed relative to 18S rRNA level. Thus, reduced plastid rRNA levels in *rfc3* grown in the Suc3 media might result from increased cytosolic rRNA levels. Interestingly, the leaf variegation phenotype of *variegated2* (*var2*), which is defective in plastid-localized metalloprotease, is enhanced by mutations in cytosolic ribosomal protein genes, but this enhancement was canceled by a mutation for the plastid ribosomal protein L24 (RPPL24) gene (Wang et al., 2017). This finding suggests that an appropriate balance between plastid ribosomes and cytosolic ribosomes is maintained during growth and development by an unknown mechanism and its disruption affects plastid development. Whether disruption of such a balance is linked to abnormal LR development in *rfc3* would be an interesting issue to be examined.

Common defects included not only failure of LR stem cell patterning and altered expression of root stem-cell regulatory genes (Figs 4–6), but also reduced symplasmic connectivity (Fig. 7), and cluster formation of plastids in the roots (Fig. 8). Each of these phenotypes was highly reproducible and specific, but are they mutually related in the context of LR development? The first two defects are obviously related to each other, yet further experiments are needed to understand the relationship between them. It is possible that deregulated cell proliferation in LR primordia leads to altered expression of stem cell regulatory genes or vice versa. This dilemma may be solved by generating double or higher order multiple mutants between *rfc3* and *wox5*, *plt3* and/or *plt7*.

The altered expression of root stem cell regulatory genes, however, might be rather downstream events. We previously showed that sucrose-sensitive developmental process of *rfc3* can be traced back to primary root growth rather than to LR developmental processes (Horiguchi et al., 2003). Although how plastid function ensures LR development should be investigated in a future study, it is interesting to note that plastid function (or dysfunction) influences symplasmic communication and spatial organization of the root apical meristem (Benitez-Alfonso et al., 2009; Burch-Smith et al., 2011; Stonebloom et al., 2012; Dmitrieva et al., 2017). The intercellular movement of key transcription factors, such as SHORT ROOT (SHR) and WOX5 (Nakajima et al., 2001; Pi et al., 2015), and symplasmic communication in the stem cell niche (Liu et al., 2017), are crucial for establishing and maintaining the root apical meristem. However, these regulations occur in the root apical meristem and probably in developing LR primordia, as well. In Spec-treated wild-type roots and in *rfc3-2* roots grown in Suc3 media, GFP diffusion from the primary root into LR primordia was strongly inhibited (Fig. 7), suggesting that upon impaired plastid translation in primary roots, a symplasmic boundary is generated between primary roots and LR primordia. This interpretation is consistent with the suggestion that the sucrose-sensitive event in *rfc3* is expected to reside in the primary root (Horiguchi et al., 2003). This idea also fits with the finding that symplasmic connectivity between xylem pole pericycle cells from which LRs are initiated, and neighboring cells including LR founder cells, are regulated spatially and temporally (Benitez-Alfonso et al., 2013). Manipulating callose deposition in these cells in future experiments could help examine whether reduced symplasmic connectivity and plastid translation is linked to regulating LR development.

If reduced symplasmic communication is relevant to abnormal LR development, the last question concerning plastid translation and LR development in this study is how impaired plastid translation affects the processes between plastids and other cellular compartments. In this regard, plastid clusters formed adjacent to the nucleus are interesting if we consider chloroplast stromule-nucleus contacts are proposed to have a signaling function during the immune response (Caplan et al., 2015). However, plastid clusters found in this study and immunity-induced stromules are not very similar to each other, except that both of them have a close association with the nucleus (Fig. 8). In addition, stromule-nucleus contact might have a more passive role (Erickson et al., 2017). Therefore, developmental meanings of plastid cluster and its association with the nucleus, if any, should be carefully investigated in the future.

Several reports suggest that retrograde signaling is triggered by impaired plastid translation and it links to developmental processes (Moschopoulos et al., 2012; Tameshige et al., 2013; Mateo-Bonmatí et al., 2015), although these mutations affect leaf adaxial-abaxial polarity and signaling mechanisms behind this phenotype are unclear. Interestingly, *root initiation defective2* (*rid2*) shows both leaf polarity and LR defects; *rid2* roots form abnormal LRs similar to stubby LRs found in *rfc3* when they are cultured in root-inducing medium (Konishi and Sugiyama, 2003). The *rid2* mutation also affects leaf adaxial-abaxial polarity and produces pointed leaves (Ohbayashi et al., 2011; Matsumura et al., 2016). *RID2* encodes a cytosolic ribosome biogenesis factor (Ohbayashi et al., 2011). Surprisingly, *rid2* phenotypes are suppressed by a second mutation in NO APICAL MERISTEM, ARABIDOPSIS TRANSCRIPTION ACTIVATION FACTOR1/2 and CUP-SHAPED COTYLEDON2 (NAC) transcription factor gene, *SUPPRESSOR OF RID TWO1* (*SRIW1*)/*ANAC082* (Ohbayashi et al., 2017). Although speculative, phenotypic commonality between mutants defective in plastid and cytosolic ribosome related genes might have overlapping signaling components. In this perspective, the LR phenotypes of the temperature-sensitive mutants *rid1-1* and *shoot redifferentiation defective2-1* (*srd2-1*), which form nodule-like LRs at 28°C (Ohtani et al., 2010, 2013), are strikingly similar to those of *rfc3*. The pattern of *SCR* and *WOX5* expression in the root meristem of *rid1-1* (Ohtani et al., 2013) also resembles that of *rfc3-2*. However, *RID1* and *SRD2* function in pre-mRNA splicing in the nucleus (Ohtani et al., 2010, 2013) and are unlikely to be directly associated with plastid gene expression. Elucidating commonality and differences in the subcellular and molecular phenotypes among *rfc3 rid1*, *rid2* and *srd2* will further advance our understanding of the role of plastid function in LR stem cell patterning as well as regulatory mechanisms that respond to defects of a broad category of gene expression systems. Finally, although the developmental functions of housekeeping genes have often been overlooked, there are increasing examples of housekeeping gene mutations that produce specific developmental defects (Tsukaya et al., 2013). As our study illustrates, there may be additional unidentified molecular processes that involve housekeeping genes.

## MATERIALS AND METHODS

### Plant materials and growth conditions

L*. er* and Col-0 were used as wild-type Arabidopsis plants. *rfc3-1* and *rfc3-2* (Horiguchi et al., 2003), *rap-1*/SAIL_1223_C10 (Kleinknecht et al., 2014), *rbf1-2*/SALK_058490 (Fristedt et al., 2014), *prps17-1* (Romani et al., 2012), *WOX5p::GFP* (Blilou et al., 2005), *SCRp::GFP:SCR/scr-3* (Nakajima et al., 2001) and QC markers (Sabatini et al., 1999) were described previously. The *WOX5p::GFP* and QC25 were backcrossed with L. *er* and/or *rfc3-2* at least three times. Plants were cultured on a solid medium leaning backward a few degrees from the vertical position under a long-day condition (16 h light, 8 h dark) at 22°C to observe the root phenotypes. The solid medium included 0.5× Murashige and Skoog (MS) salts, 0.05% (w/v) MES-KOH pH 5.7, sucrose at an indicated concentration, and 0.5% (w/v) gellan gum. Plants were cultured on rock wool under the long-day condition to observe leaf phenotypes. Stock solutions of plastid ribosome inhibitors dissolved in distilled water or ethanol were added to autoclaved 0.5× MS Suc3 medium before solidification. Spectinomycin dihydrochloride pentahydrate (Wako, Osaka, Japan), kanamycin sulfate (Wako), and streptomycin sulfate (Wako) were used as plastid ribosome inhibitors and were added to 0.5× MS Suc3 or Suc0.5 at various concentrations.

### Plasmid construction and plant transformation

The cDNA sequences were subcloned into pENTR/D-TOPO with the TOPO cloning kit (Thermo Fisher Scientific) to construct *35Sp::RFC3:GFP*, *35Sp::PRPS6:GFP*, and *35Sp::MRPS6:GFP* and transferred into the pH35WG vector (G.H. and H.T., unpublished data) using Gateway LR clonase II (Thermo Fisher Scientific). The transit-peptide sequence of the *RecA* gene was subcloned into pENTR/D-TOPO (called pENT-RecA-TP) after referring to a previous study (Köhler et al., 1997) to construct *35Sp::RecA-TP:CFP*. The 35S promoter cloned into pDONRP4P1R and pENT-RecA-TP was reacted with R4pGWB543 (Nakagawa et al., 2008) using LR clonase II plus (Thermo Fisher Scientific). A genomic *RFC3* fragment from the 4044-bp 5′-region upstream of the region just before the *RFC3* stop codon (in total, 5709 bp) was cloned into pENTR/D-TOPO (Thermo Fisher Scientific) to construct *RFC3g:GFP* and then transferred into a Gateway binary vector containing a promoter-less *GFP* cassette, pHWG (G.H. and H.T., unpublished data). The *SUC2* promoter and *sGFP* fragment were amplified by polymerase chain reaction (PCR) and reacted with the linearized binary vector pSMAH621 (Kubo et al., 2005) using the In-Fusion HD cloning kit (TaKaRa Bio, Shiga, Japan) to construct *SUC2p::sGFP*. *35Sp::RecA-TP:CFP* and *RFC3g:GFP* were introduced into wild-type L. *er* and/or *rfc3-2* and *SUC2p::sGFP* was introduced into Col by floral dipping using *Agrobacterium tumefaciens* strain ASE or C58C1. A *SUC2p::sGFP* line was crossed with *rfc3-2* three times. We used medium containing 0.5× MS salts pH 5.7, Suc3 or Suc0.5, 0.3% (w/v) gellan gum, 20 mg l^−1^ hygromycin, and 500 mg l^−1^ cefotaxime to select T1 plants. Primer sequences used for construction are shown in Table S2.

### Quantitative RNA analyses

RNA samples were extracted from roots of 8-days post-sown seedlings frozen in liquid nitrogen using the TRI reagent (Molecular Research Center, Cincinnati, OH, USA) according to the manufacturer's instructions. SuperScript III Reverse Transcriptase (Thermo Fisher Scientific) and its accessory primers were used for reverse transcription. Oligo(dT) primers and random hexamers were used for the expression analysis of nuclear-encoded genes (except for cytosolic rRNAs) and of plastid-encoded genes and cytosolic rRNAs, respectively. Quantitative PCR analysis was performed using GoTaq qPCR Master Mix (Promega, Madison, WI, USA) with an Applied Biosystems 7500 Fast Real-Time PCR system (Thermo Fisher Scientific). Relative expression levels of nuclear-encoded genes and plastid-encoded genes were calculated with the ΔΔCT method, and normalization was based on *ACT2* and *ct18S* expression, respectively. The primer sequences are shown in Table S2.

### Phylogenetic analysis

The sequences analyzed were identified with BLAST searches at several websites: TAIR (https://www.arabidopsis.org/index.jsp), Phytozome (ver. 9.1; http://www.phytozome.net/), GreenPhyl (ver. 3; http://www.greenphyl.org/v3/), and NCBI (http://www.ncbi.nlm.nih.gov/). Sequences of *Marchantia polymorpha RFC3*, *PRPS6* and *MRPS6* homologs were identified from RNA-seq data generated in the Kohchi laboratory (http://marchantia.info/genome/index.php/). The sequences identified were aligned with the MAFFT program (Katoh et al., 2002; Katoh and Standley, 2013) and sites containing >50% gaps were excluded with the trimAl tool (Capella-Gutierrez et al., 2009). Phylogenetic trees were prepared according to the maximum likelihood (ML) method with the JTT model, and analyzed with the RAxML program (Stamatakis, 2006). Alignment files were converted to PDF files with the ClustalX program (http://www.clustal.org/clustal2/), and phylogenetic trees were displayed with MEGA6 software (Tamura et al., 2013).

### Transient expression analysis using leaf mesophyll protoplasts

Leaf mesophyll protoplasts of 1-month-old plants grown on rock wool were prepared according to the Tape-Arabidopsis sandwich method (Wu et al., 2009). Vectors were isolated from transformed *Escherichia coli* DH5α with the JetStar Plasmid Midi Kit (Veritas Genetics, Danvers, MA, USA) and transfected by the PEG method, based on a previous report (Yoo et al., 2007). After a 16-h culture at 22°C, transfected protoplasts were mounted with WI solution (0.5 M D-mannitol, 20 mM KCl, 4 mM MES-KOH pH 5.7). MRPS6:GFP-transfected protoplasts were treated with 50 µM MitoTracker Red (Thermo Fisher Scientific) to stain mitochondria on a slide for 30–60 min at 22°C and washed with WI solution before mounting the protoplasts. Stained and unstained protoplasts were observed with an LSM 710 laser scanning microscope (Carl Zeiss, Zena, Germany) (GFP, λ_ex_=488 nm, λ_em_=493–556 nm, chlorophyll autofluorescence, λ_ex_=633 nm, λ_em_=647–721 nm, and MitoTracker Red, λ_ex_=561 nm, λ_em_=568–614 nm).

### Chlorophyll fluorescence measurements

Chlorophyll fluorescence parameters were measured with a MINI-PAM (pulse-amplitude modulation) portable chlorophyll fluorometer (Walz, Effeltrich, Germany) in ambient air at room temperature (25°C). Minimum chlorophyll fluorescence at the open PSII center (*F*o) was determined by measuring light at 0.05–0.1 µmol photons m^–2^ s^–1^. A saturating pulse of white light (800 ms, 3000 µmol photons m^–2^ s^–1^) was applied to determine the maximum fluorescence level at the closed PSII center in the dark (*F*m). Maximum fluorescence level at the closed PSII center (*F*m′) and the steady-state fluorescence level (*F*s) in actinic light (120 µmol photons m^–2^ s^–1^) were determined. The maximum PSII activity and PSII (Φ_PSII_) quantum yield were calculated as (*Fm*−*F*o)/*F*m and (*F*m′−*F*s)/*F*m′, respectively.

### Immunoblot analysis

Aerial parts of 10-days post-sown seedlings of L. *er* and *rfc3-2* grown on rock wool were homogenized in protein extraction buffer [20 mM Tris-HCl, pH 6.8, 2% (w/v) sodium dodecyl sulfate, 24% (v/v) glycerol] on ice using a hand-operated homogenizer, and sediment was removed by two centrifugation steps. The concentrations of extracted protein samples were determined with a DC Protein Assay Kit (Bio-Rad) before adding 1/10 volume of 1 M dithiothreitol. Approximately 100 ng and 3.5 µg total protein from extracts were electrophoresed on 10% (w/v) isocratic polyacrylamide gels containing SDS, transferred to Amersham Hybond LFP 0.2 PVDF membranes (GE Healthcare), reacted with each antibody, and detected with Amersham ECL Prime (GE Healthcare) and the ImageQuant LAS 4000mini (GE Healthcare) for immunoblotting of D1 and the other proteins. Antibodies to anti-RPS6 and anti-RPL5 detected both of the paralogous ribosomal proteins (RPS6A/B and RPL5A/B, respectively). After detection, the membranes were stained by Amido Black solution [1 mg ml^−1^ Amido Black, 45% (v/v) methanol, and 10% (v/v) acetic acid].

### Detection of ribosomal proteins in polysomal fractions

Total cell extracts were prepared from 5 ml of frozen ground seedlings and suspended in polysome extraction buffer (PEB) containing 200 mM Tris-HCl (pH 9.0), 200 mM KCl, 50 mM ethylene glycol tetraacetic acid, 100 mM MgCl_2_, 6 mM β-mercaptoethanol, 2 mM phenylmethylsulfonyl fluoride (PMSF), 1% (v/v) Triton X-100, 1% (v/v) Brij 35, 1% (v/v) Tween-40, 1% (v/v) NP-40, 2% (v/v) polyoxyethylene 10 tridecyl ether, 1% deoxycholic acid, 50 µM cycloheximide, 50 µM chloramphenicol, and 1 mg ml^−1^ heparin. Procedures for sucrose density gradient sedimentation analyses were carried out according to the methods described by Natori et al. (2007) and Nanamiya et al. (2010). After removing cell debris by centrifugation (12,000 ***g***, 15 min, 4°C), aliquots of the supernatant were layered onto 15–60% (w/v) sucrose density gradients in Buffer I (20 mM Tris-HCl pH 7.6, 10 mM magnesium acetate, 100 mM ammonium acetate, 6 mM βmercaptoethanol, and 2 mM PMSF) and centrifuged (65,000 ***g***, 17.5 h, 4°C, Hitachi P40ST rotor). Samples were taken with a Piston Gradient Fractionator (BioComP, Fredericton, NB, Canada), and absorbance profiles were monitored at 254 nm using a Bio-mini UV Monitor (ATTO, Tokyo, Japan). Polysomal fractions were subjected to sodium dodecyl sulfate-polyacrylamide gel electrophoresis and immunoblot analysis (Fig. S12). Rabbit antiRPS6 and RPL5 antibodies were raised against the synthetic peptides, DTEKPRMRGPKRASKIRC and VEATGEDFSVEPTDSRRC, respectively.

### Transmission electron microscopy

*A. thaliana* roots were cut into 2–3-mm pieces and fixed with 4% (w/v) paraformaldehyde and 2% (v/v) glutaraldehyde in 50 mM sodium cacodylate buffer (pH 7.4) overnight at 4°C. They were post-fixed with 1% (w/v) osmium tetroxide in 50 mM cacodylate buffer for 2 h at 21°C. After dehydration in a graded methanol series [25, 50, 75, 90, and 100% (v/v)], the samples were infiltrated with increasing concentrations of Epon812 resin [propylene oxide: Epon812=3:1, 1:1, 1:3, and 100% (v/v)] and embedded. Ultrathin sections (80 nm) were cut with a diamond knife on an ultramicrotome (Leica EM UC7, Leica Microsystems, Buffalo Grove, IL, USA) and mounted on formvar-coated copper grids. The ultrathin sections were stained with 4% (w/v) uranyl acetate followed by lead citrate solution and examined with a transmission electron microscope (JEM-1400; JEOL Ltd., Tokyo, Japan) at 80 kV.

### Other procedures

We mounted the samples treated with or without 10 µg ml^−1^ propidium iodide (PI) for 5 min on a slide to observe GFP and CFP patterns in root cells and leaf mesophyll cells. The samples were observed with the LSM 710 (GFP only, λ_ex_=488 nm, λ_em_=493–598 nm, GFP/Chl, λ_ex_=488 nm/633 nm, λ_em_=493–598 nm/647–721 nm, GFP/PI, λ_ex_=488 nm, λ_em_=493–556 nm/593–719 nm, CFP/PI, λ_ex_=405 nm/514 nm, λ_em_=454–581 nm/593–719 nm, and CFP/GFP, λ_ex_=405 nm/488 nm, λ_em_=454–581 nm/493–598 nm). The modified pseudo-Schiff-PI (mPS-PI) method was performed as described previously (Truernit et al., 2008) with some changes. Roots were fixed with 50% (v/v) ethanol and 10% (v/v) acetic acid overnight or for several days at 4°C and treated with 80% (v/v) ethanol for 3 min before washing and treatment with 1% (w/v) periodic acid solution. The treated samples were stained with 100 µg ml^−1^ PI for 2 h, cleared in a clearing solution (chloral hydrate:glycerol:water=8:1:2) overnight at 4°C, mounted, and scanned with the LSM 710 (λ_ex_=514 nm, λ_em_=566–719 nm). The roots were soaked in a β-glucuronidase (GUS) solution [500 mg /l 5-bromo-4-chloro-3-indolyl-β-D-glucuronic acid, 100 mM NaPO_4_, pH 7, 3 mM potassium ferricyanide, 10 mM EDTA, 0.1% (v/v) Triton X-100] overnight at 37°C for GUS staining, washed in 70% (v/v) ethanol twice, and mounted in clearing solution. We used a Leica M165FC equipped with a DFC300FX camera (Leica) as a stereomicroscope, and a Leica DM2500 equipped with a DFC420C camera (Leica) as a differential contrast interference microscope. Adjustments and format conversions of confocal images were performed using Fiji software (Schindelin et al., 2012). Statistical analyses and drawing graphs were performed using the ‘R’ software (R Development Core Team, 2010) or Excel 2008 for Mac (Microsoft). Captions, diagrams, and coloring for explanation were superimposed on the figures using Adobe Illustrator CS4 software (Adobe Systems).

## Supplementary Material

Supplementary information
