# Supplementary material for: Plastid translation is essential for lateral root stem cell patterning in Arabidopsis thaliana
Source: Biol Open. 2018 Jan 24;7(2):bio028175. doi: 10.1242/bio.028175 (PMC5861355; doi:10.1242/bio.028175)
Supplement: Supplementary information [file biolopen-7-028175-s1.pdf]

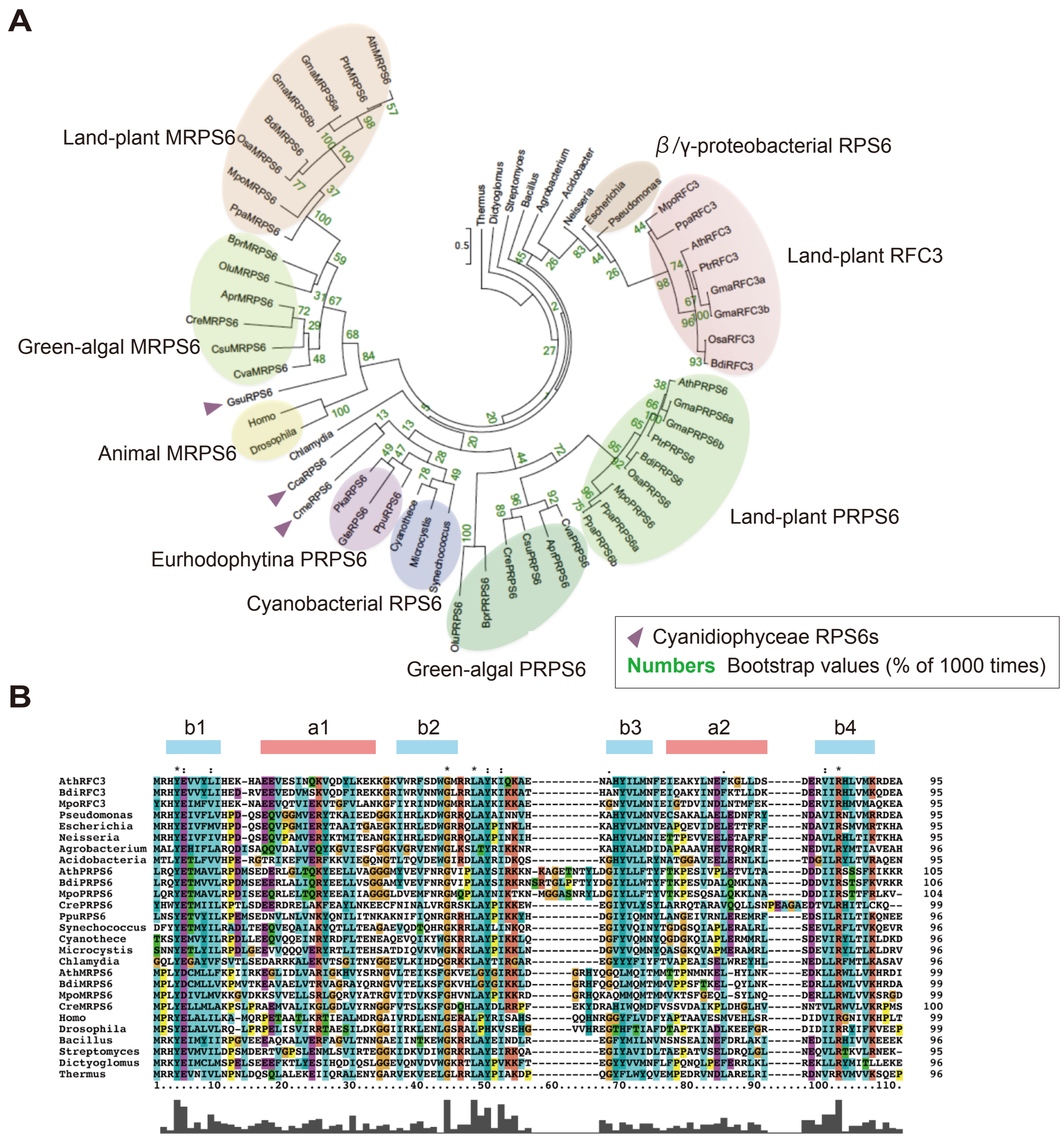

Figure S1. Phylogenetic tree and alignment of bRPS6 family proteins

Figure S1. Phylogenetic tree and alignment of bRPS6 family proteins. (A) An unrooted phylogenetic tree of bRPS6 family proteins by ML method with JTT model. Numbers indicate bootstrap values (% of 1000 times). Arrowheads show Cyanidiophyceae species. Detailed name of each species and accession numbers of used sequences are shown in Table S1. (B) Alignment of the bRPS6 domain. Ath, *Arabidopsis thaliana*; Bdi, *Brachypodium distachyon*; Mpo, *Marchantia polymorpha*; Cre, *Chlamydomonas reinhardtii*; Ppu, *Porphyridium purpureum*; animal mitochondria Homo, *Homo sapience* mitochondrial RPS6; Drosophila, *Drosophila melanogaster* mitochondrial RPS6; the others, RPS6s from bacterial species (refer to Table S1). “a1” and “a2” indicate the first and second alpha helices and “b1” to “b4” indicate the first to fourth beta sheets, respectively.

Biology Open • Supplementary information

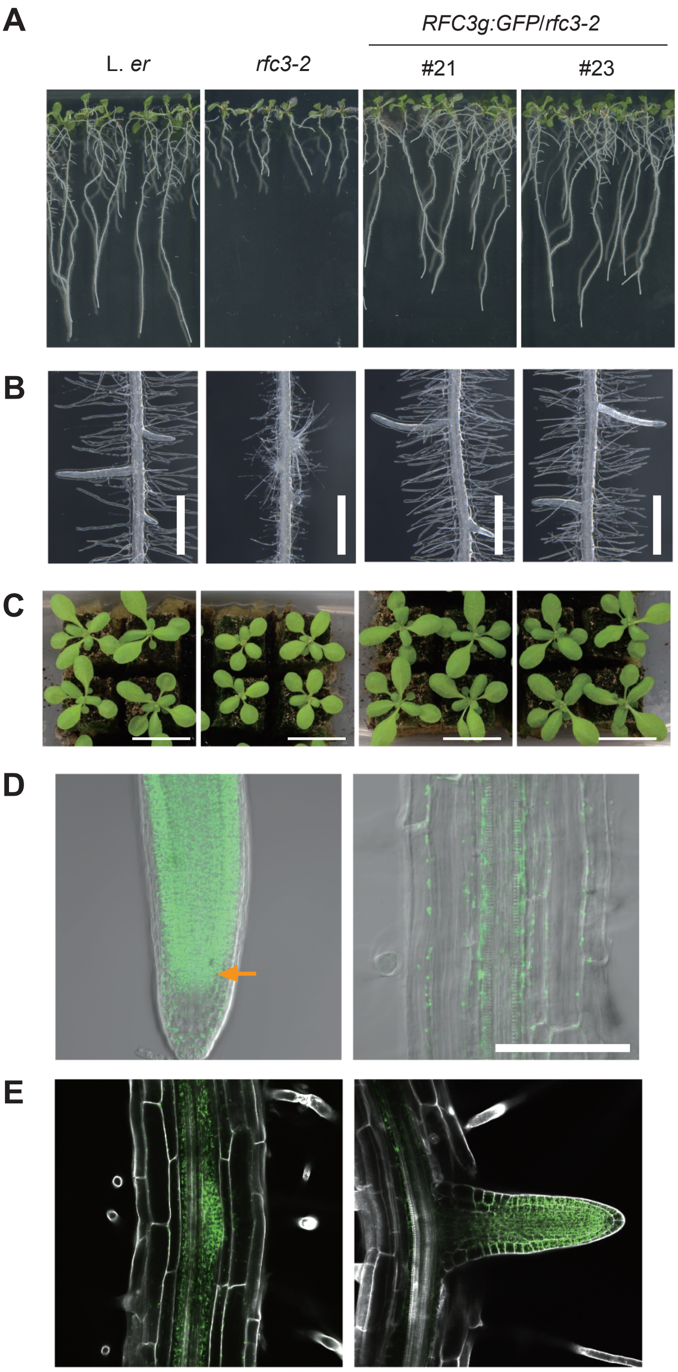

Figure S2. Subcellular localization of RFC3:GFP in stable lines of *RFC3g:GFP/rfc3-2.* (A-C) Phenotype of primary roots (A), LRs (B) and shoots at 20-days-post-sowing (C) in rescued lines of *RFC3g:GFP/rfc3-2*. (D-E) RFC3:GFP pattern (green) in primary roots (D), an LR primordium (E, left panel) and an LR (E, right panel). Propidium iodide (PI)-stained cell wall is shown as white in (E). An arrow in D indicates QC cells. An arrowhead in (E) indicates an LR primordium. Scale bars equal 1cm (A, C), 1 mm (B) and100 μm (D, E).

Biology Open • Supplementary information

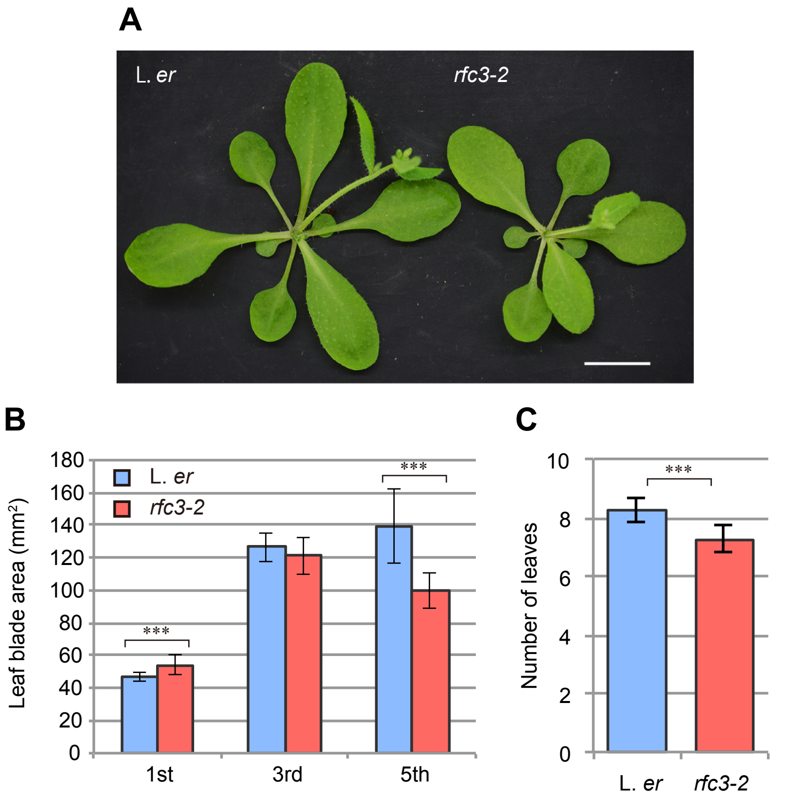

Figure S3. Shoot phenotypes of *rfc3-2*. (A) Shoots of L. *er* and *rfc3-2* grown for 23 days. Bar indicates 1cm. (B) Leaf balde area. (C) Number of leaves per plant. Data are mean ± s.d. (n = 12). Statistical analyses in (B) and (C) were carried out using the paired Student' s *t*-test and statistical significance is indicated by triple asterisks ($p < 0.001$).

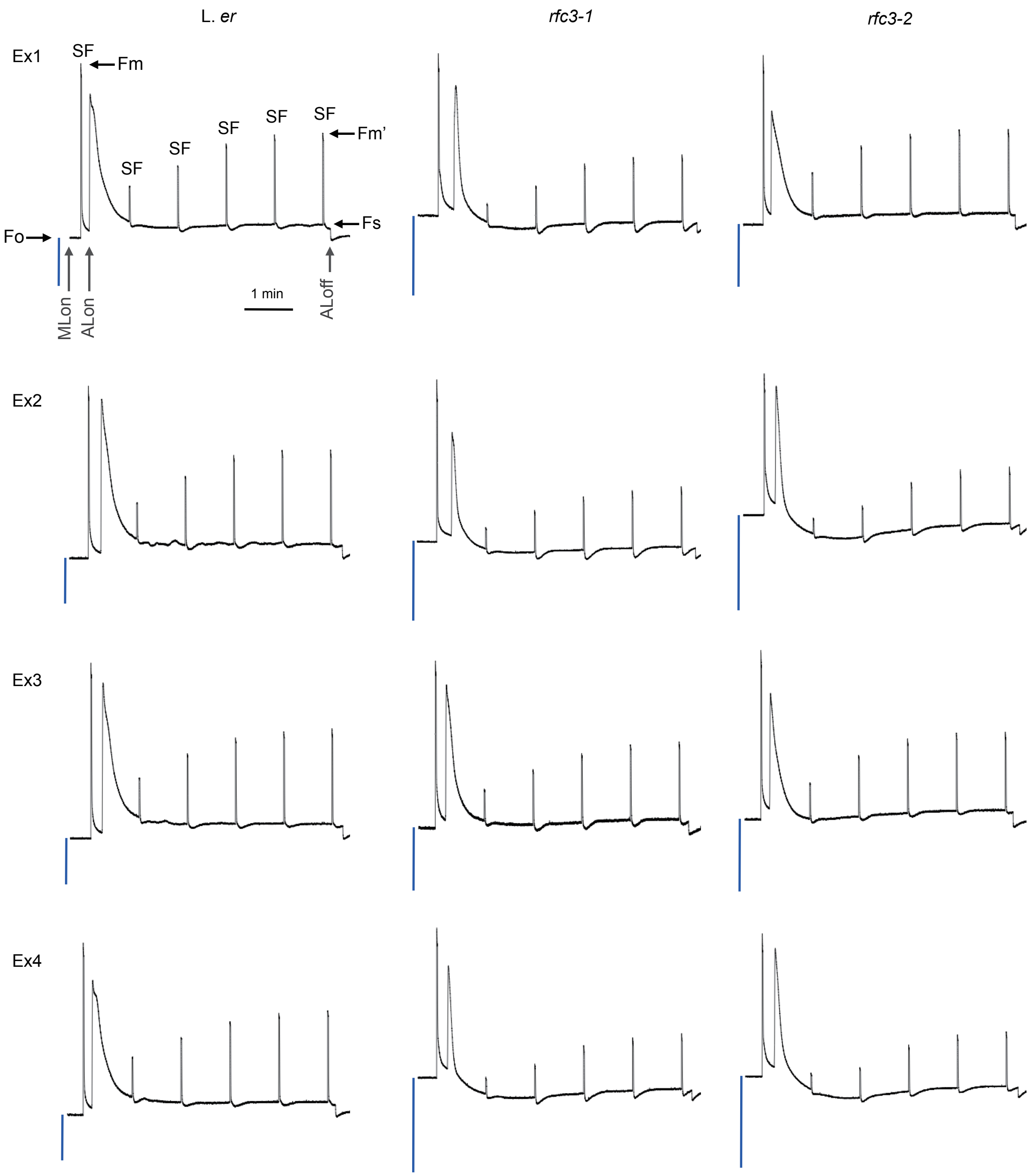

Figure S4. Chlorophyll fluorescence induction pattern of *rfc3* alleles. Results from four independent experiments (Ex1-Ex4). Vertical blue bars represent the Fo levels. Chlorophyll fluorescence was analyzed under the measuring light (ML). Photosynthesis was induced by actinic light (AL, 120 μmol photons m^{-2} s^{-1}). Saturating flushes (SF) were applied every 1 min to monitor Fm and Fm' .

Biology Open • Supplementary information

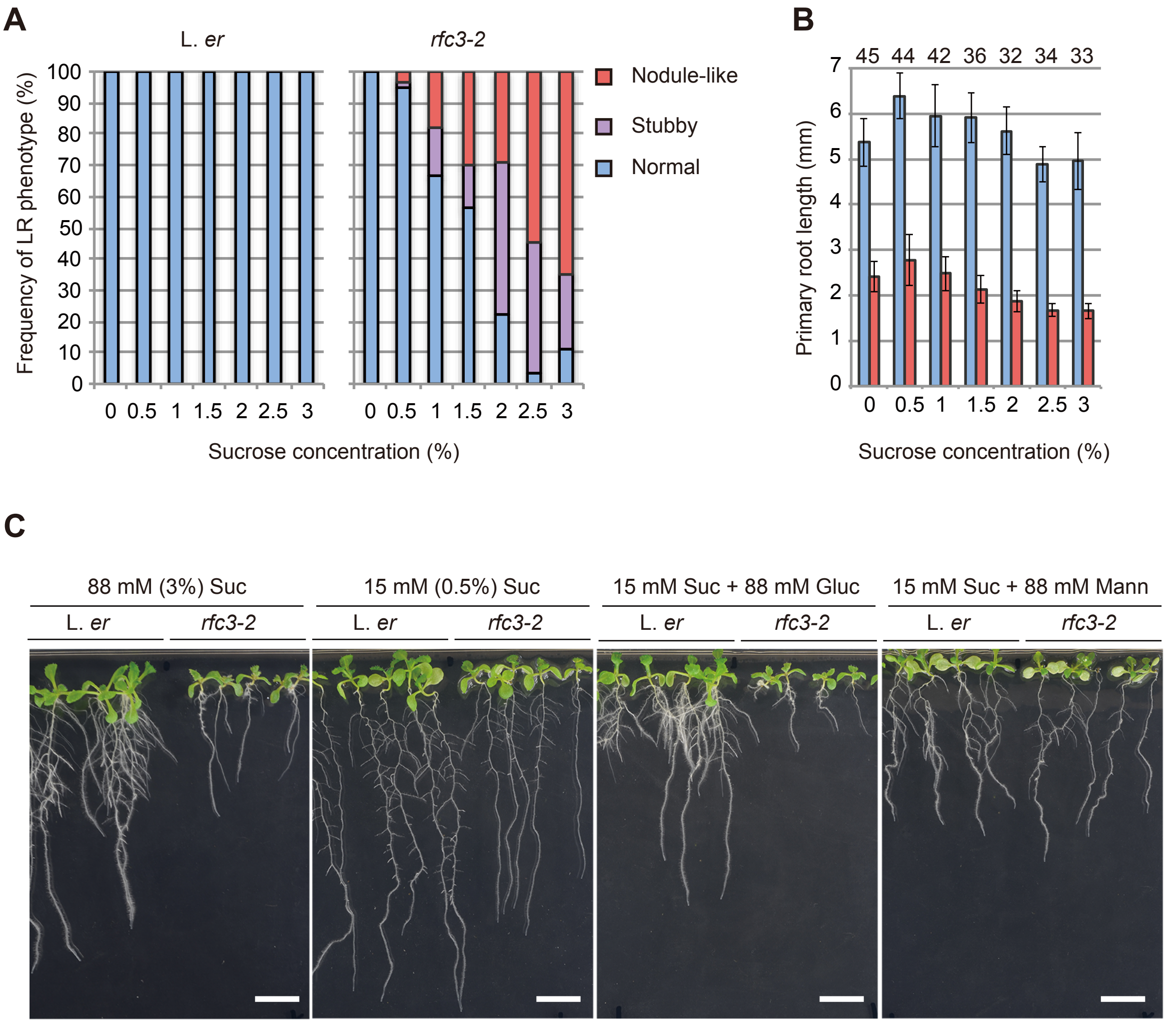

Figure S5. Effects of sugars on LR development. (A) Frequencies of normal, stubby, and nodule-like LRs in L. *er* and *rfc3-2*. (B) Primary root length. (C) L. *er* and *rfc3-2* grown in media containing various sugar conditions. L. *er* (n = 20) and *rfc3-2* (n = 30) primary roots grown for 8 days were examined. In (A), LRs beyond stage VI and all LRs were scored. In (B), data are presented as mean ± s.d. and relative primary root lengths of *rfc3-2* to L. *er* are also indicated on the top of graph. Scale bars in (C) indicate 1 cm.

Biology Open • Supplementary information

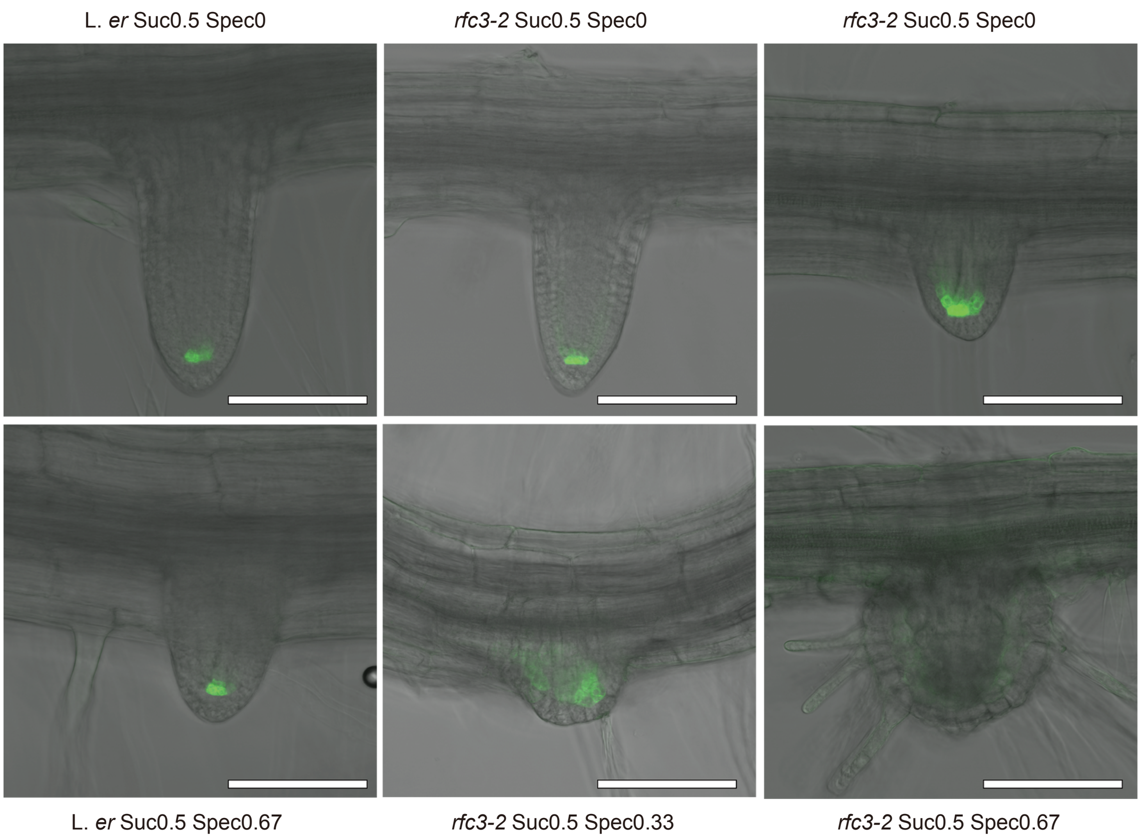

Figure S6. *WOX5p::GFP* pattern in LR primordia of *rfc3-2*. L. *er* and *rfc3-2* were grown in media containing 0.5% sucrose and Spec at indicated concentration (mg L^{-1}). GFP fluorescence images were merged with differential interference contrast images. Bars indicate 100 μm.

Biology Open • Supplementary information

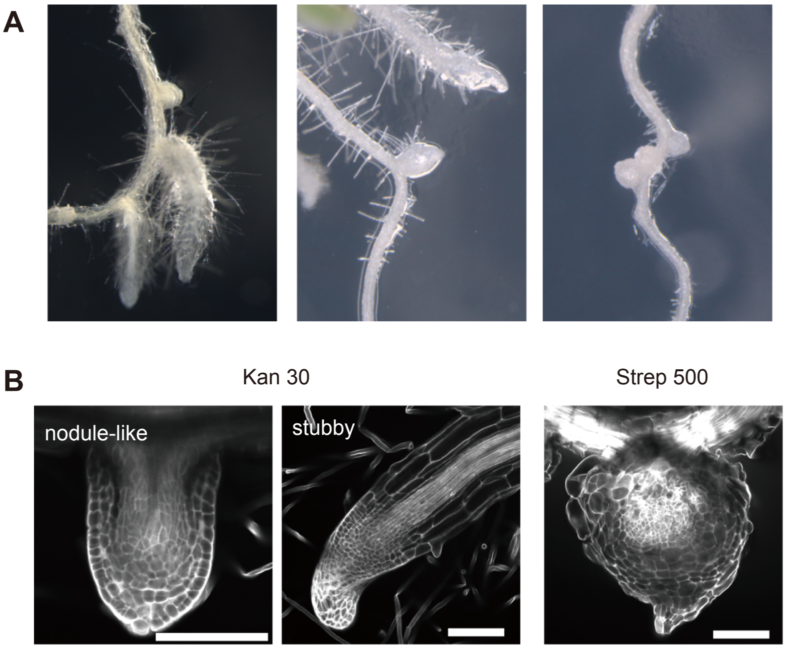

Figure S7. The effect of plastid translation inhibitors on LR development. (A) LR phenotype of wild-type L. *er* plants treated with plastid translation inhibitors. Yellow arrowheads, nodule-like LRs; red arrowheads, stubby LRs. The numbers described in Figures are the concentration of each inhibitor (mg L^{-1}). (B) LR phenotype of kanamycin (Kan)-treated and streptomycin (Strep)-treated plants stained by mPS-PI methods. Scale bars equal 1 mm (A) and 100 µm (B).

Biology Open • Supplementary information

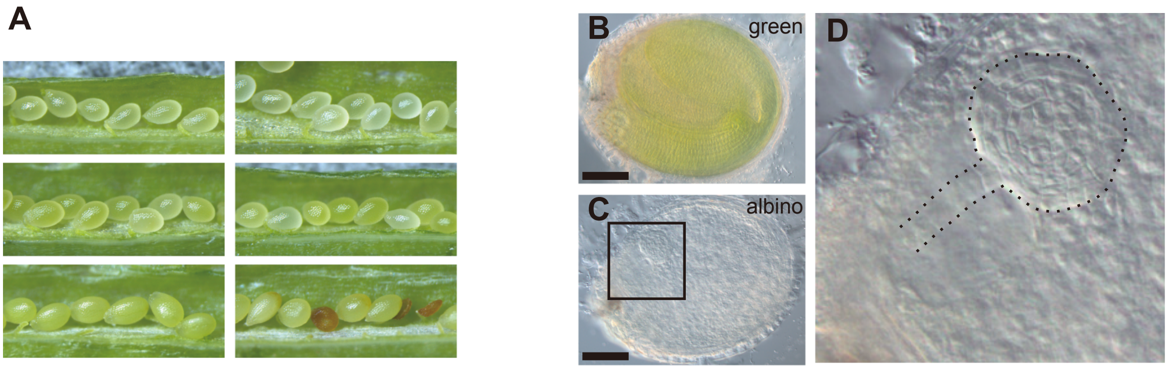

Figure S8. Genetic interaction between *rfc3* and *prps17.* (A) Developing ovules from immature to mature fruits. (B-D) Ovlules containing a green embryo (B) and an albino embryo (C), respectively. An enalraged image of a square in (C) is shown in (D). Scale bars equal 1 mm (A) and 100 µm (B, C).

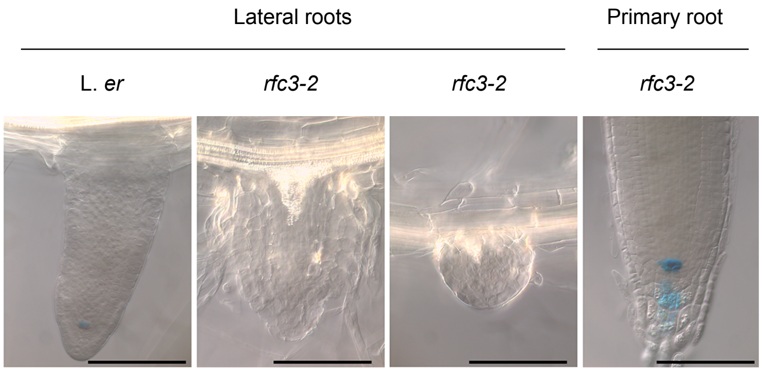

Figure S9. Expression of QC25 marker. QC25 reporter lines in L. *er* and *rfc3-2* backgrounds were subjected to GUS staining. Bars indicate 100 μm.

Biology Open • Supplementary information

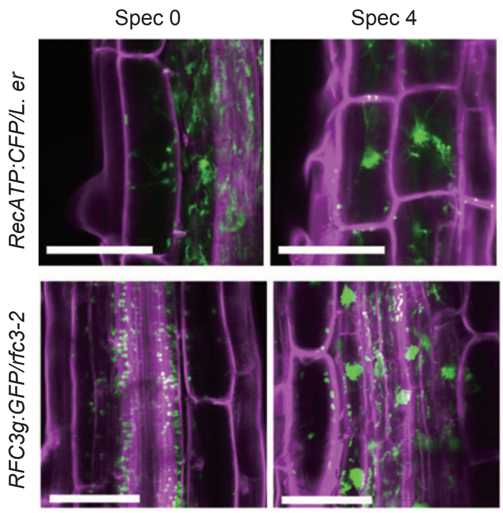

Figure S10. Plastid marker aggregations in mature root cells of Spec-treated L. *er* plants. The patterns of stroma markers of root plastids in the mature region of PRs. The fluorescence pattern of *35Sp::RecA-TP::CFP* or *RFC3g::GFP* are shown in green and propidium iodide is shown in magenta. Scale bars, 50μm.

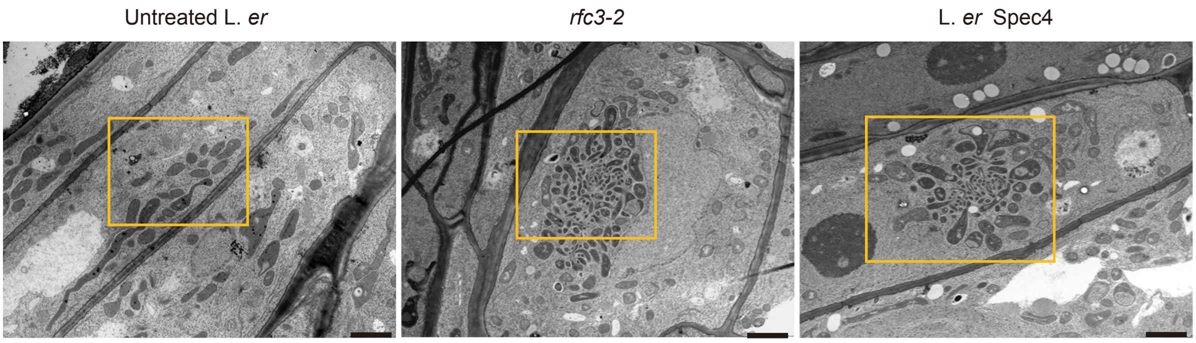

Figure S11. Enormous plastid clusters in mature root cells of *rfc3-2* and Spec-treated L. *er* plants. Ultrastructure of root plastids in untreated L. *er*, *rfc3-2* and 4 mg L^{-1} Spec-treated L. *er* plants. Figure 8B corresponds to magnified views of orange squares of this figure. Scale bars equal 2 µm.

Biology Open • Supplementary information

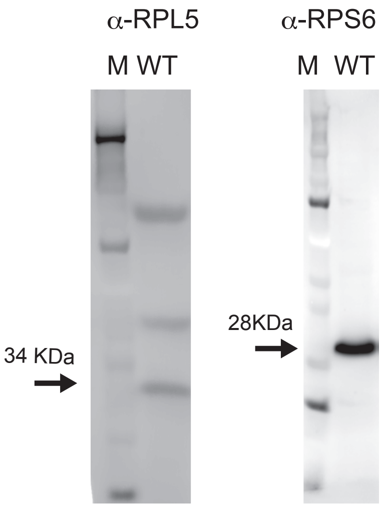

Figure S12. Detection of RPL5 and RPS6. Polysomal fractions from wild type (WT) seedlings were isolated and subjected immunoblot analysis. Both anti-RPL5 and -RPS6 antibodies detected proteins at the estimated molecular weights of RPL5 and RPS6, respectively. M, Molecular weight marker.

Table S1. Information of RPS6 sequences for phylogenetic analysis

Click here to Download Table S1

Table S2. Information of primers used in this study

Click here to Download Table S2

Biology Open • Supplementary information
